# CnRed: Efficient, Marker-free Genome Engineering of *Cupriavidus necator* H16 by Adapted Lambda Red Recombineering

**DOI:** 10.1021/acssynbio.4c00757

**Published:** 2025-02-24

**Authors:** Simon Arhar, Johanna Pirchner, Holly Stolterfoht-Stock, Karin Reicher, Robert Kourist, Anita Emmerstorfer-Augustin

**Affiliations:** 1 Austrian Centre of Industrial Biotechnology, acib GmbH, 8010 Graz, Austria; 2 Institute of Molecular Biotechnology, Graz University of Technology, NAWI Graz, 8010 Graz, Austria; 3 BioTechMed-Graz, 8010 Graz, Austria

**Keywords:** *Cupriavidus necator*, recombineering, lambda Red, genetic engineering, phytase, electroporation

## Abstract

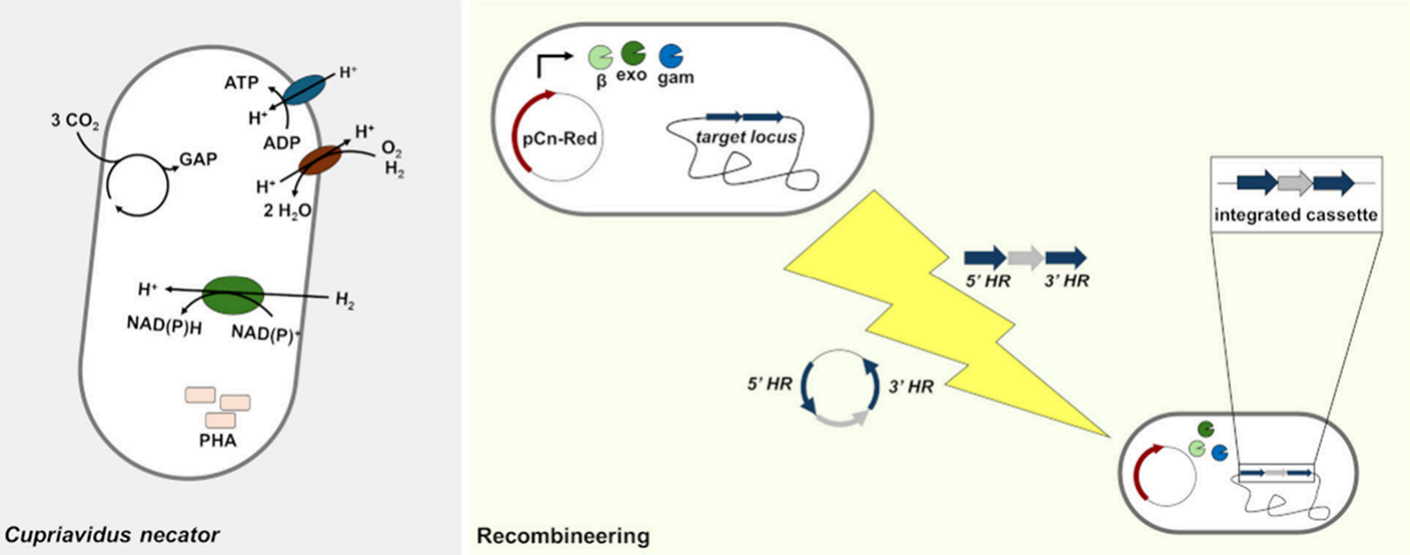

Due to its ability to utilize carbon dioxide, native intracellular accumulation of bioplastic precursors, and a high protein content, the bacterium *Cupriavidus necator* offers potential solutions for social problems tackled by modern biotechnology. Yet, engineering of high-performing chemolithotrophic production strains has so far been hindered by the lack of adequate genome editing methods. In this work we present the establishment of a lambda Red recombineering system for use in *Cupriavidus necator* H16. In combination with electroporation as DNA delivery system, it enables an efficient and fast gene deletion methodology utilizing either suicide plasmids or, for the first time, linear PCR product. The novel lambda Red system was validated for the modification of three different genomic loci and, as a proof-of-concept, ultimately utilized for stable genomic integration of *Escherichia coli* phytase gene *appA* into the *phaC1* locus. A Cre/*loxP* system further enabled efficient marker recycling. The combination of a minimal transformation protocol with lambda Red recombineering and a Cre/*loxP* system offers a robust, freedom-to-operate synthetic biology tool in an increasingly important bacterial production host. This approach simplifies and accelerates genome engineering in *C. necator* and is expected to significantly enhance future strain development efforts.

## Introduction

*Cupriavidus necator* H16, previously known as *Ralstonia eutropha* H16, is a Gram-negative soil bacterium with a highly versatile metabolism. Its remarkable ability to utilize CO_2_ as carbon source and accumulate substantial amounts of bioplastic precursors has led to growing interest in applying *C. necator* as a sustainable production platform. As model organism for chemolithotrophic growth on CO_2_,^1−3^*C. necator* employs the Calvin–Benson–Bassham (CBB) cycle while generating the necessary energy through hydrogen oxidation.^4^ Additionally, *C. necator* thrives on various compounds present in organic waste streams, including organic acids, alcohols, aromatic compounds, and certain sugars such as fructose and N-acetylglucosamine.^5^ Fructose is metabolized via the Entner–Doudoroff pathway, which serves as an alternative to glycolysis and is characterized by the intermediate keto-deoxy-phosphogluconate and its cleavage by the enzyme aldolase Eda.^6^ Notably, *C. necator* has the capacity to accumulate biodegradable polyhydroxyalkanoates (PHA), including polyhydroxybutyrate (PHB), as a storage compound. This capability has gained significant attention due to its potential for bioplastics production. Recent studies have demonstrated that *C. necator* H16, when cultivated under chemolithoautotrophic conditions, can accumulate PHB, comprising up to 79% of its total biomass.^7^ Consequently, it is unsurprising that PHA production in *C. necator* is attracting increasing industrial interest, with ongoing efforts aimed at further optimizing this process. Furthermore, the general necessity to decrease CO_2_ emissions and the current dependency on fossile ressources by the chemical industry, make *C. necator* an attractive platform for sustainable synthesis of compounds besides PHA. Among them are bulk chemicals like isopropanol,^8^ value-added products like terpenoids^9,10^ and polymers like cyanophycin.^11^ To enhance yields of such compounds, researchers frequently consider utilizing PHA-negative strains with a defective *phaCAB* operon. This strategic approach allows for the diversion of carbon toward targeted production, highlighting the versatile potential of *C. necator* in synthetic biology and biotechnology.^8,9,12^

Due to the increasing interest in *C. necator*, a growing number of genetic resources have become available. However, metabolic engineering of *C. necator* is still limited by available tools.^13^ Currently, targeted genetic manipulation is predominantly achieved through the conjugation of unstable suicide plasmids and the native recombination machinery. As established by Simon et al. in 1983, the typical steps involve cloning the desired DNA into a mobile plasmid, transforming it into an *E. coli* S17-1 donor strain, and facilitating the conjugative transfer of the plasmid into the recipient strain.^14,15^ Overall, this conjugation process is labor-intensive and time-consuming, often exhibiting low efficiency, which limits its application in high-throughput settings. As an alternative, electroporation has emerged as a more time-efficient method for introducing DNA into *C. necator*. Early protocols demonstrated functionality but suffered from inconsistent transformation efficiencies, heavily dependent on the construct being used.^16,17^ Recent advancements have addressed these limitations through modifications of the *C. necator* restriction-modification system and the development of suitable vectors.^18−21^ With significant progress in the electroporation of *C. necator*, the next crucial step is to utilize this method for genome editing. Initial attempts in this direction involved constructing a CRISPR/Cas9-based editing tool. While the published CRISPR technology yielded some promising results in *C. necator*, the applied methods have shown limited versatility and have resulted in prolonged experimental durations, leading to a scarcity of examples demonstrating their practical application.^18,22^

An alternative and powerful approach to genome editing is offered by the use of phage-derived recombineering methods.^23^ These methods are well-established in model organisms like *E. coli* and stand out as versatile, efficient and robust tools for genetic engineering. Unlike CRISPR/Cas9 strategies, they further provide the advantage of a well-established freedom-to-operate framework. The lambda Red recombineering strategy has proven to be particularly successful^23,24^ and works by enhancing homologous recombination through the annealase (Beta), the exonuclease (Exo), and an inhibitor of the bacterial host’s RecBCD system (Gam).^25^ The corresponding genes are typically controlled by an inducible promoter on an easily curable, low-copy-number plasmid.^26,27^ The delivery system for the recombinant or mutated gene is usually provided on linear DNA fragments, or occasionally on circular plasmids. When circular plasmids are used, suicide plasmids are often employed in an “in–out” strategy, where the gene of interest in the plasmid is first recombined into the bacterial target locus, followed by resolution of the cointegrate.^28^ This method is frequently applied with vectors that cannot replicate under the conditions used for cointegrate selection, or by using origins of replication with low stability.^32^ In cases where more stable plasmids are used, Red recombineering can also be applied to retrieve genes from the bacterial chromosome onto the plasmid backbone, such as by exchanging a selection marker flanked by homologous regions with the target sequence.^29^ However, due to the genetic instability associated with the use of plasmids, the use of linear DNA fragments, typically generated by PCR amplification, is generally preferred in recombineering approaches.^27^

The genes used in lambda Red recombineering originate from bacteriophage lambda, making this system particularly effective in *E. coli*. However, it has also been successfully adapted for a variety of alternative bacterial hosts, including *Vibrio cholerae* and *Pseudomonas aeruginosa* (reviewed by ref (23)). The extent of modifications required often correlates with the phylogenetic distance of these bacteria from *E. coli*. In many cases, adaptations to the expression machinery are necessary to enable effective application in the host organism.^30−32^ In this context, we report the development of a straightforward lambda Red recombineering system specifically designed for *C. necator*, utilizing recently established low-copy-number plasmids.^21^ We demonstrate the system’s effectiveness by successful inactivation of three different genes using either suicide plasmids or linear DNA fragments, achieving editing efficiencies of at least 64% and 74%, respectively. Furthermore, we employed the lambda Red system for the stable integration of the *appA* gene, which encodes a phytase, into the *phaC1* locus. To enhance this system further, we implemented marker recycling using the Cre/*loxP* system, encoded on an electroporation plasmid, allowing for the efficient generation of marker-free strains.

## Results

### Design of a Recombineering System for Gene Knockouts in *C. necator*

**Figure 1:**
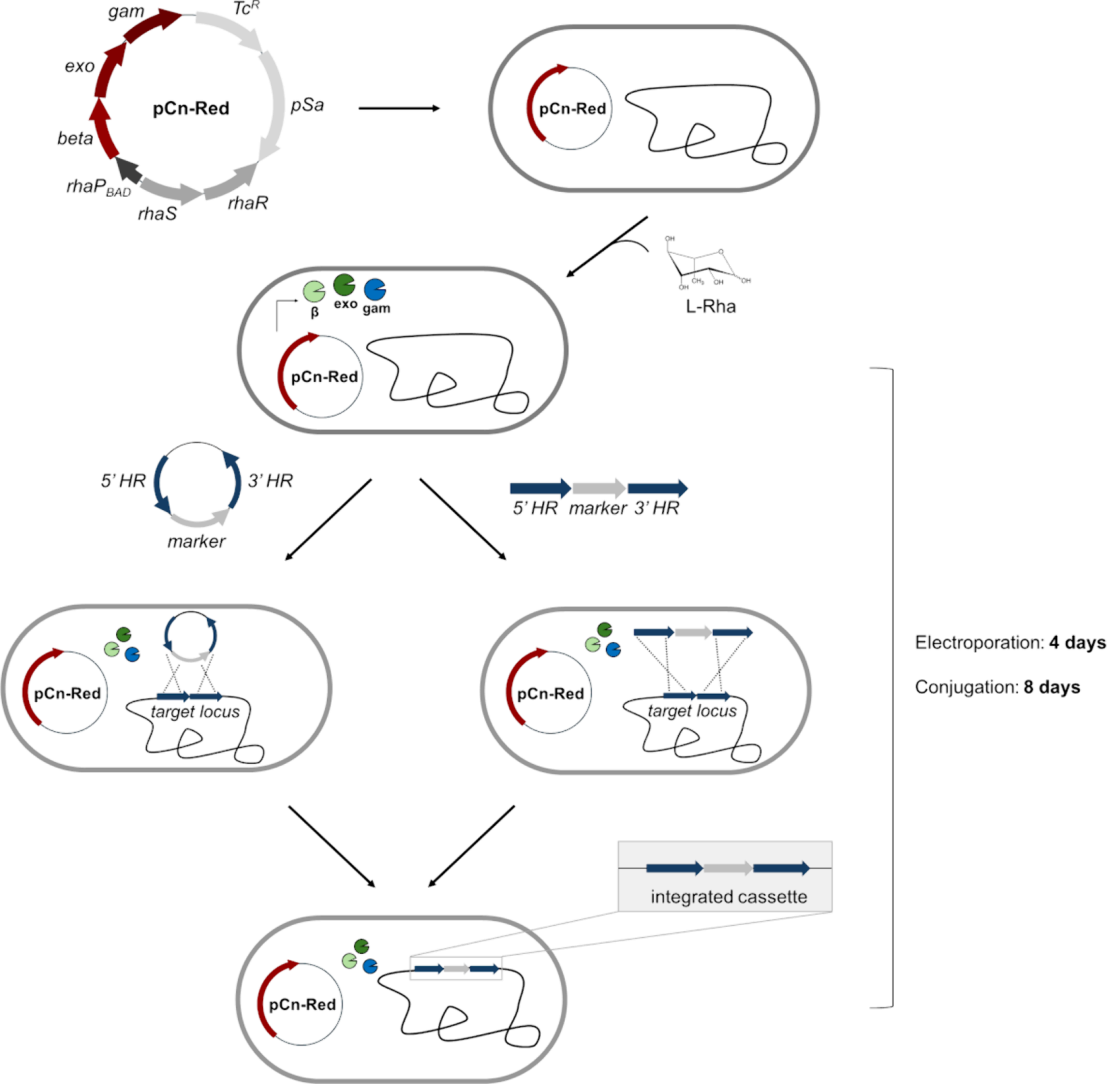
Schematic overview of the lambda Red recombineering system adapted for *C. necator*. Cells are transformed with a plasmid for the expression of lambda *beta*, *exo*, and *gam*, with induction achieved through rhamnose. The strain producing lambda Red is further transformed with either a suicide plasmid or linear double stranded DNA, both containing the marker flanked by sequences homologous to the target locus. Lambda Red facilitates the replacement of the target locus via double crossover. The use of electroporation as vector delivery method reduces the time to obtain a recombinant clone to half compared to the standard conjugation-based methods, from 8 to 4 days.

To facilitate fast and easy genome modifications in *C. necator*, three different recombineering systems were tested and compared: the lambda *red*(23,24) and *recET*(33) operons, as well as the *C. necator recA* gene.^34,35^ All systems were expressed from plasmids harboring the rhamnose-inducible promotor from *E. coli*, which was reported to be tightly controllable in *C. necator*.^36,37^ To facilitate polycistronic expression of the lambda *red* phage and *recETE. coli* genes, a reportedly strong ribosome binding site for *C. necator* was used.^38^ The plasmid contains the low-copy number pSa origin of replication^21^ and a tetracycline resistance cassette for plasmid maintenance (Figure 1). Gene expression of the recombineering systems was induced during preparation of electrocompetent cells, following established practices in other bacterial systems.^39,40^ A model strain containing an *eGFP* expression cassette integrated into the *phaC1* locus was used to facilitate easy detection of successful integration of repair plasmids and linear fragments. Double-stranded DNA vectors were designed to replace the genomic *eGFP* cassette in the model strain with a kanamycin resistance cassette through recombineering (Figure 1). The cassettes were introduced either via a suicide plasmid containing the relatively unstable pLO3 origin of replication,^41^ or as a PCR product comprising the resistance cassette and homologous regions corresponding to the target locus. Initially, different induction periods were tested for the production of recombineering systems. Prolonged induction negatively impacted cell growth (Figure S1), and transformation efficiencies (Figure S2, A). While the induction of the *recET* system had the highest impact on cells growth, transformation of neither plasmid nor linear repair fragments yielded viable transformants (Figure S2, A). Overall, only lambda Red achieved sufficient transformation rates, particularly at reduced induction times (Figure S2, A and B). To mitigate these issues and to minimize the risk of unwanted background mutations, as reported in *E. coli* K-12,^42^ rhamnose was added only during the final cell density doubling prior to harvest in all subsequent experiments. Editing efficiencies remained consistent at ∼70–80% for plasmids and 90–100% for linear DNA fragments, regardless of whether lambda Red or *C. necator* RecA was used.

Additionally, we tested varying concentrations of the suicide plasmid (100, 300, and 600 ng) and linear DNA fragments (0.5, 1, and 2 μg) under optimized lambda Red recombineering conditions to assess their impact on transformation and editing efficiencies (Figure S3). While increasing DNA concentrations did not significantly affect transformation efficiency (Figure S3, A), lower concentrations resulted in slightly reduced editing efficiency in both cases (Figure S3, B). Based on these findings, we conducted subsequent recombineering experiments using 300 ng of suicide plasmid and 1 μg of linear repair fragments.

**Figure 2:**
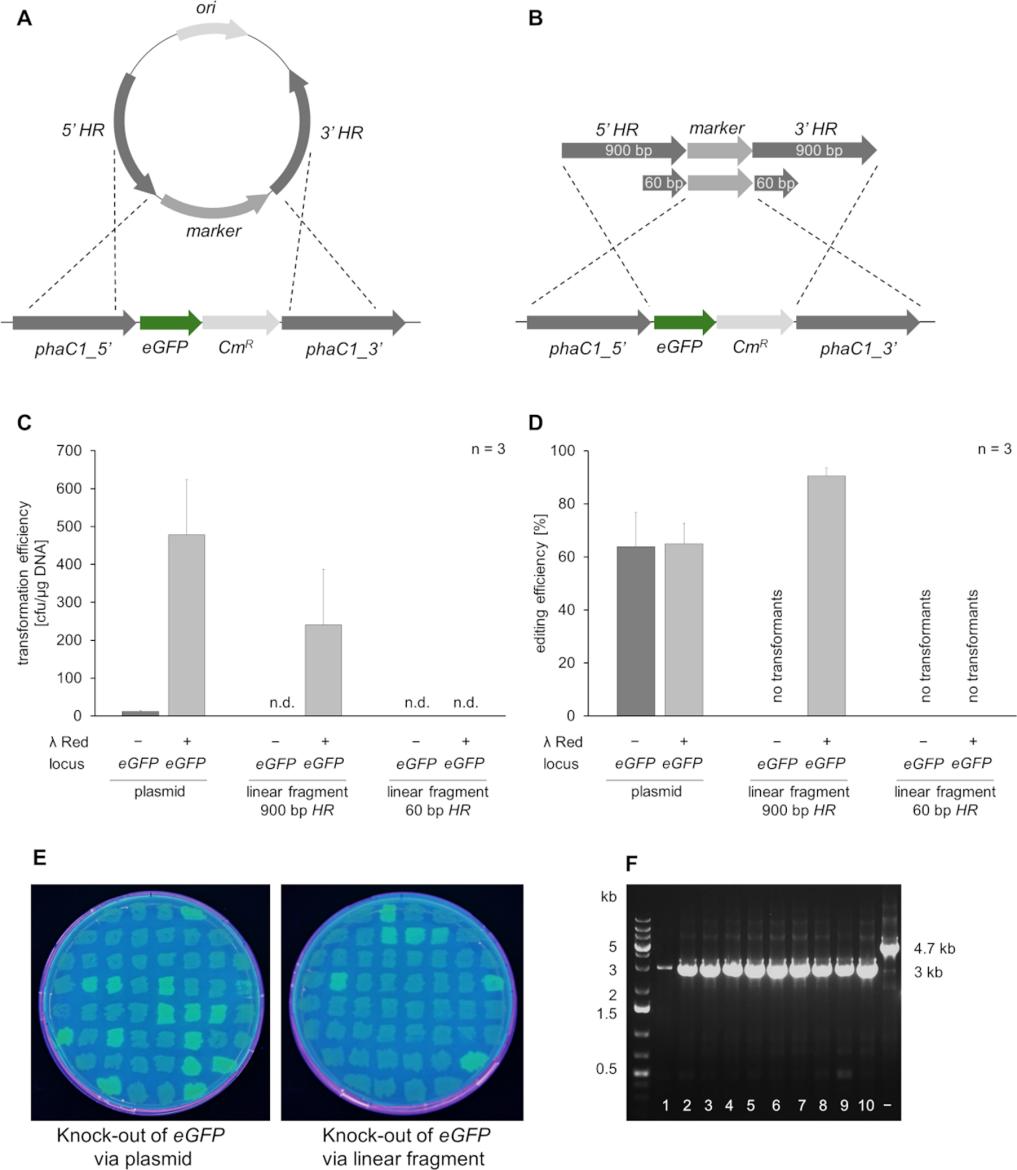
Suicide plasmid and linear PCR product derived DNA vectors efficiently replace genomic *eGFP* through lambda Red-aided recombination. (A) A schematic representation of a suicide plasmid designed for targeting the *eGFP* locus is shown. The integration cassette includes a selection marker flanked by homologous sequences. Dotted lines indicate the expected sites of a double crossover. (B) Schematic of linear double-stranded DNA generated by PCR, with 900 bp and 60 bp homologous arms, showing the expected regions for crossover at the target locus. (C) Electroporation rates for suicide plasmid and the PCR product were calculated for cells with and without lambda *red* expression. Data from three transformations using two separate batches of electrocompetent *C. necator* cells are presented. (D) Editing efficiencies for each construct targeting *eGFP* were determined based on the screening of 50 colonies per transformation for the expected fluorescence phenotype. (E) An example of phenotypic screening: For transformants that lost fluorescence a replacement of the eGFP locus is indicated. (F) Verification of the correlation between phenotype and genotype was conducted via colony PCR of 10 clones per transformation. The *eGFP* locus (−) produced a 4.7 kb PCR product, while replacement with the Kan^R^ cassette yielded a 3 kb PCR product.

Cells transformed with circular and linear fragments containing 900 or 60 bp homologous regions were screened for positive integration by plating on kanamycin-containing agar plates and assessing the loss of fluorescence (Figure 2, A and B). The advantage of using shorter, 60 bp homologous regions in linear integration cassettes is that these constructs can be easily generated by PCR amplification of an existing (e.g., Kan^R^) cassette, thus facilitating and streamlining the process of generating target knockouts in *C. necator*. For the suicide plasmid, the use of the heterologous recombineering system increased transformation rates from approximately 12 cfu/μg DNA to 4.8 × 10^2^ cfu/μg DNA (Figure 2, C). No transformants were observed with either of the linear PCR products (containing 900 or 60 bp of homologous regions) in cells that did not express lambda Red. However, in cells expressing lambda Red, approximately 2.2 × 10^2^ cfu/μg DNA were achieved for constructs containing 900 bp of homologous regions. When the homologous regions were shortened to 60 bp, constructs failed to recombine (Figure 2, C and D) and this could not be improved by increasing the amount of repair DNA from 1 μg to 2 μg (data not shown). Overall, these data, obtained from three technical and two biological replicates of competent cells prepared separately on different occasions, highlight significant batch-to-batch variation and account for the high standard deviation observed (Figure 2, C). Editing efficiencies were calculated (Figure 2, D) based on the analysis of 50 clones per electroporation and whether they exhibited fluorescence under UV light (Figure 2, E). Independent of the presence of lambda Red, an editing efficiency of around 64% was found with the suicide plasmids (Figure 2, D). In contrast, the linear fragment transformed into the strain expressing lambda *red* exhibited an editing efficiency of 90%. To verify the correlation between phenotype and genotype, ten clones per transformation that showed the expected phenotype were analyzed by colony PCR. All clones tested by colony PCR exhibited the correct 3 kb PCR product, confirming the successful replacement of the target locus with the Kan^R^ cassette (Figure 2, F).

### Efficient Knockout of Native *proC* and *eda* Loci Using Suicide Plasmids and Linear Fragments

After establishing a proof of principle for the recombineering system with the *eGFP* locus, the approach was further validated by targeting the native *C. necator* loci of *proC* and *eda*, which code for a pyrroline-5-carboxylate reductase and a keto-deoxy-phosphogluconate aldolase, respectively. Knockout of *proC* results in proline dependency, whereas knockout of *eda* impairs growth on sugars due to its role in the Entner–Doudoroff pathway, producing easily detectable phenotypes.^11,43^

**Figure 3:**
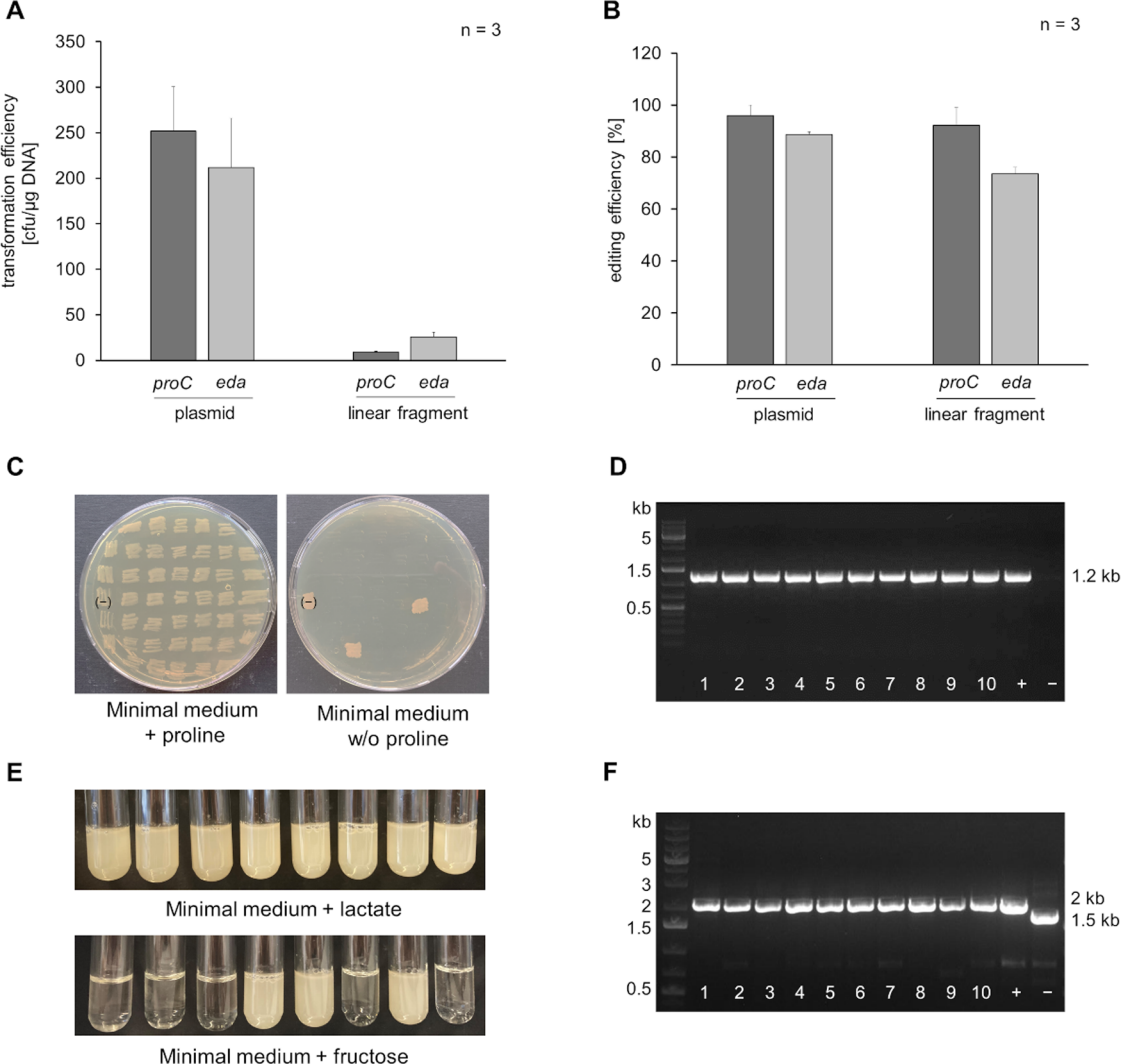
The lambda Red system facilitates efficient replacement of the *C. necator proC* and *eda* genes using either suicide plasmids or linear vector DNA. (A) Transformation rates for suicide plasmids and linear PCR-derived vector DNA, targeting *proC* and *eda* were assessed. (B) Editing efficiencies were calculated based on phenotypic screening. (C) Transformants targeting the *proC* locus were restreaked on minimal medium with and without proline to verify successful deletions. Clones that grew exclusively on proline-supplemented medium were indicative of *proC* replacement. (D) Genotypic verification of the Δ*proC* mutants was performed using PCR. No PCR product was observed for the wild-type *proC* locus (−) with the selected primers, while the integration of the Kan^R^ cassette resulted in a 1.2 kb PCR product. (E) Δ*eda* mutants were identified by differential growth on minimal medium containing either lactate or fructose. Transformants that grew exclusively on lactate were considered Δ*eda* mutants. (F) Genotypic verification of the Δ*eda* mutants was conducted via PCR. The wild-type *eda* locus (−) and the integrated Kan^R^ cassette produced PCR products of 1.5 kb and 2 kb, respectively. Positive controls (+) were included from previously confirmed mutant strains.

*C. necator* H16 cells expressing the lambda *red* system were transformed with suicide plasmids and linear fragments designed to target the two loci via homologous recombination. Targeting the *proC* locus, the transformation rates were approximately 2 × 10^2^ cfu/μg DNA for the suicide plasmid and 9 cfu/μg DNA for the linear PCR product (Figure 3, A). Putative Δ*proC* mutants were identified by screening transformants on minimal agar plates with and without 0.2% proline. Clones that grew exclusively on proline-supplemented agar plates were considered recombinants and were used to calculate editing efficiencies (Figure 3, B and C). The suicide plasmid achieved an editing efficiency of around 96%, while the linear fragment yielded 92% (Figure 3, B). Phenotype-genotype correlation was confirmed by analyzing 10 clones exhibiting the correct phenotype through colony PCR. Primers were designed to produce a PCR product only if the Kan^R^ cassette was correctly integrated at the locus (Figure 3, D). Similarly, cells transformed with constructs targeting the *eda* locus were selected on kanamycin agar containing 2% lactate as an alternative carbon source to fructose. The transformation rate for the suicide plasmid was again approximately 2 × 10^2^ cfu/μg DNA, while the linear fragment achieved 25 cfu/μg DNA (Figure 3, A). To identify potential Δ*eda* mutants, the transformants were selected on minimal medium containing 2% fructose or 2% lactate. Clones growing exclusively on lactate-containing medium were considered Δ*eda* knockouts (Figure 3, E). The suicide plasmid achieved an editing efficiency of around 89%, while the linear fragment yielded 74% (Figure 3, B). Genotype verification was performed by colony PCR with a 500 bp shift of the amplicon expected upon replacement of the *eda* gene with the kanamycin resistance cassette.

### Recombineering-Based Introduction of Marker-Free Phytase Expression in *C. necator*

**Figure 4:**
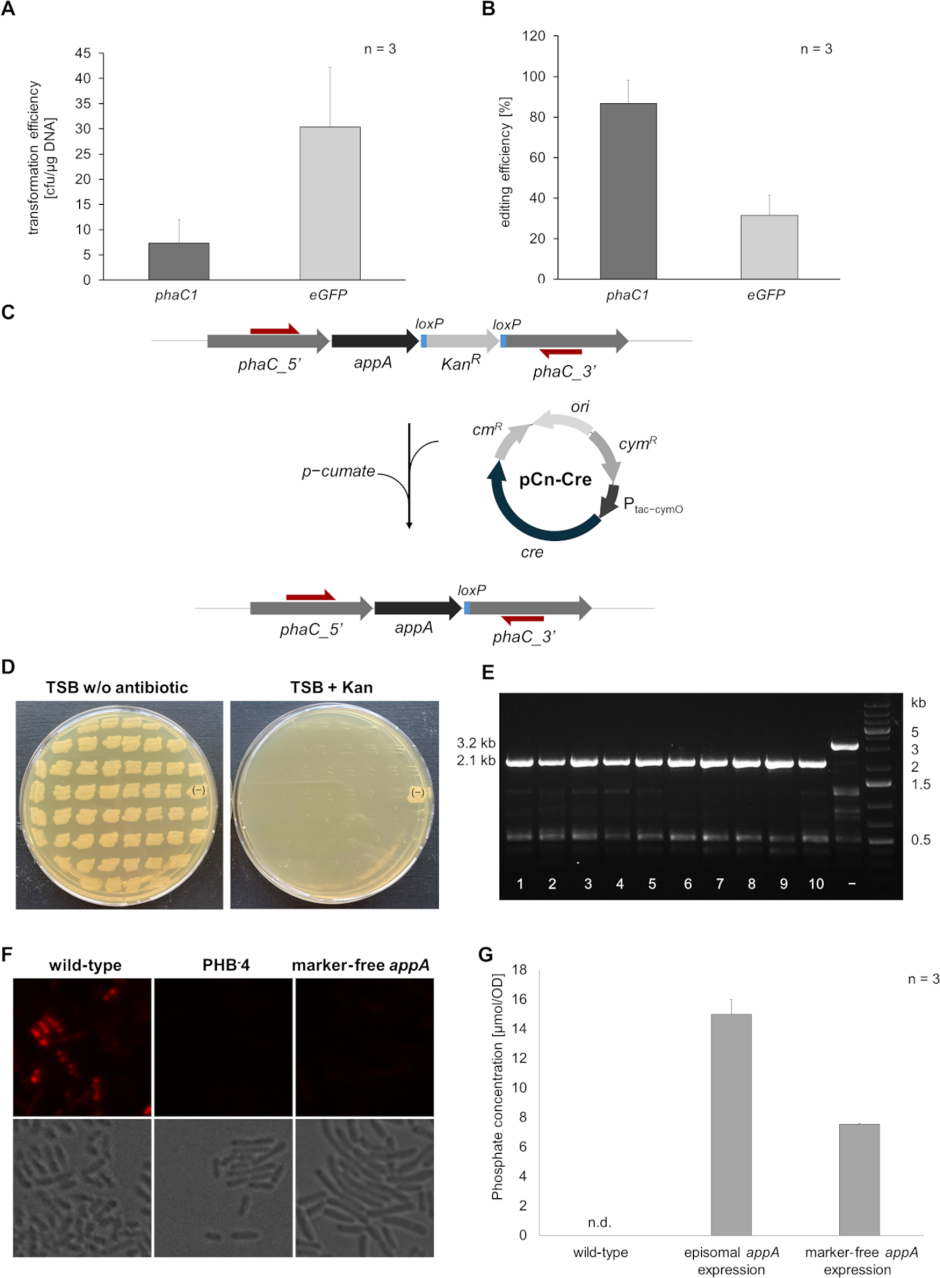
An *appA* expression cassette was integrated into the *phaC1* locus of *C. necator*, followed by marker recycling and strain characterization. (A) Transformation rates and (B) editing efficiencies for the integration of the *appA* cassette into *C. necator* wild-type and the *eGFP* expression strain were determined as previously described. (C) A Cre/*loxP* recombination system, with Cre recombinase encoded on the pCn-Cre plasmid and controlled by a cumate-inducible promoter, was used for marker recycling. (D) Screening for marker-free mutants was conducted by restreaking on agar plates with and without kanamycin, where transformants that grew only on TSB without antibiotics were considered marker-free. (E) Genotypic verification of marker recycling was performed via colony PCR, revealing a 3.2 kb PCR product before the removal of Kan^R^, while successful marker recycling resulted in a 2.1 kb PCR product. (F) PHA depletion, a consequence of successful *phaC1* disruption by *appA*, was verified by Nile red staining followed by fluorescence microscopy. The marker-free *appA* expression strain was compared with *C. necator* H16 wild-type and *C. necator* PHB^–^4, with each image representing a 12.2 × 12.2 μm section. (G) Phytase activities were evaluated by measuring phosphate released from phytic acid after 15 min of incubation with cell-free lysate, including samples from *C. necator* wild-type and a control strain expressing *appA* from a plasmid.

To broaden the application of the lambda Red recombineering system, the integration of the *E. coli appA* gene, encoding a phytase, into the *C. necator* genome was investigated. Two strains were used: the wild-type H16 and an *eGFP* strain, which has an *eGFP* expression cassette integrated into the *phaC1* locus. The integration module was designed with an *appA* expression cassette, including a kanamycin resistance marker flanked by *loxP* sites, along with homologous regions flanking the cassette for recombination into the *phaC1* locus (Figure 4).

When targeting the *phaC1* locus with the suicide plasmid harboring *appA*, an unexpected observation was made. Amplification of the DNA inserted between the homologous arms of the plasmid revealed PCR products of varying sizes. These corresponded not only to the size of the *appA* expression cassette but also to the wild-type locus that was intended to be replaced in the genome (Figure S4). Contrary to expectations, the cells did not eliminate the plasmid but instead stably maintained it for an extended period, as confirmed by colony PCR (Figure S5). Plasmid isolation and sequencing did not yield analyzable results, raising concerns that an active lambda Red system might lead to genetic instability through recombination events, potentially causing target and recombinant genes to switch between loci. To minimize this risk, we decided to exclusively use linear PCR products for *appA* integration. Transformation rates varied depending on the strain. The wild-type strain exhibited approximately 7 cfu/μg DNA, while the *eGFP* strain showed around 30 cfu/μg DNA (Figure 4A). In the *eGFP* strain, successful *appA* integration was visually confirmed by the loss of fluorescence, whereas in the wild-type strain, screening was performed via colony PCR (Figures S6 and S7). The editing efficiency for the linear fragment targeting *phaC1* was around 87%, nearly three times higher than the 30% efficiency observed for the *eGFP* locus (Figure 4, B).

To recycle the kanamycin resistance marker cointegrated with the *appA* expression cassette, a small plasmid encoding the Cre recombinase under the control of a cumate-inducible tac promotor was constructed. This plasmid was transformed into engineered *C. necator* strains using a shortened protocol for preparing competent cells, adapted from methods established for *E. coli* (Figure 4, C).^44^ Transformants were selected on chloramphenicol plates containing *p*-cumate. To identify those that had lost the antibiotic resistance marker, transformants were restreaked on plates with and without kanamycin (Figure 4, D). All tested clones exhibited a loss of kanamycin resistance after two rounds of testing. Correct marker recycling was confirmed in all clones by colony PCR (Figure 4, E).

To ensure the removal of the pCn-Red plasmid, which expresses the lambda *red* system in *C. necator* H16, a plasmid curing protocol was implemented. *C. necator* cells were incubated in minimal medium for 48 h over two incubation cycles, allowing sufficient time for natural plasmid loss. Afterward, the cells were streaked onto agar plates with and without tetracycline. None of the clones grew on tetracycline-containing plates, confirming successful plasmid loss in all restreaked clones. Similarly, the pCn-Cre plasmid, used for site-specific recombinase expression, was easily lost through the same curing steps due to its unstable origin of replication.

A marker-free *C. necator* strain with the *appA* cassette integrated at the *phaC1* locus was further analyzed for its PHB content and phytase activity. The *phaC1* gene encodes the key enzyme responsible for polymerizing hydroxyacyl-CoA monomers into PHB polymers. Therefore, disruption of *phaC1* by the *appA* expression cassette was expected to eliminate PHB production. To confirm the loss of PHB granules in the phytase-expressing strain, PHB staining with Nile red followed by fluorescence microscopy was performed (Figure 4F). *C. necator* H16 wild-type and the PHB-negative PHB^–^4 strain^45^ were used as controls. As anticipated, the wild-type *C. necator* H16 strain accumulated significant PHB granules, indicated by intracellular fluorescence signals under the tested cultivation conditions (Figure 4G). In contrast, both the PHB-negative PHB^–^4 strain and the marker-free *appA* expression strain showed no detectable fluorescence signals. Phytase activity was assessed by measuring phosphate release from phytic acid incubated with cell lysate. The marker-free genomic *appA* expression strain was compared with wild-type *C. necator* H16 and an episomal *appA*-expressing strain previously developed in our lab.^21^ The marker-free strain released approximately 8 μmol of phosphate per OD_600_ unit over a 15 min reaction period, while the episomal *appA*-expressing strain, utilizing the high-copy-number pKPRepPar_Pj5 plasmid, released around 15 μmol of phosphate per OD_600_ unit (Figure 4G).

## Discussion

Lambda Red recombineering was initially developed for *E. coli*, utilizing the native lambda phage open reading frame for the *beta*, *exo*, and *gam* genes.^46^ While this system has been successfully adapted for genetic manipulation in bacteria closely related to *E. coli*, such as the gamma-proteobacteria *Salmonella enterica*(47,48) and *Klebsiella pneumoniae*,^49,50^ its functional range is more limited in other bacterial species, particularly for dsDNA recombination.^51^ For instance, its application in Beta-proteobacteria, to which *C. necator* belongs, has been rarely reported (reviewed by ref (24)), and often requires more advanced modifications, as for example the introduction of alternative annealases.^32,40^ Furthermore, to our knowledge, a lambda infection of *C. necator* has not yet been reported, suggesting that a direct application of lambda Red recombineering may be challenging. In the lambda phage, the original *beta* and *exo* open reading frames overlap and production of Exo is therefore dependent on efficient translational coupling. Although the ATGA-DNA sequence used for coupling in the *red* operon is widespread among bacteria, its usage varies, and it remains unclear how effectively downstream proteins are produced in *C. necator*.^52^ The expression of *beta* is further dependent on a lambda phage derived ribosome binding site,^53^ of unknown strength in *C. necator*.^37^

A comparison of the *E. coli* RecET, lambda Red, and *C. necator* RecA systems revealed that the lambda Red recombineering system provided the highest cell survival, transformation, and recombineering efficiencies. To mitigate potential issues with polycistronic DNA expression and prevent read-through of the ORF, the lambda *red* expression strategy was adapted for *C. necator* by using only well-characterized genetic elements. These adjustments were implemented alongside the introduction of a functional resistance cassette and origin of replication. Known *C. necator* ribosome binding sites^38^ were placed upstream of each *red* gene, and *beta*, *exo*, and *gam* were rearranged based on their importance in the recombineering process. Since expression levels typically decrease for downstream genes in a polycistronic operon,^54^*beta*, which encodes the annealase directly responsible for recombination, was placed first. *Gam*, which is not directly involved in recombination^55^ and lacks a clear target in *C. necator* due to the absence of a complete RecBCD system, was placed last. It has been reported multiple times that prolonged exposure to lambda Red recombineering can lead to unwanted background mutations or growth defects.^41^ To minimize these risks, most plasmid-encoded recombineering systems are controlled by tightly regulatable promoters, such as the arabinose promoter, which is known for its inducible, high-level expression.^26,27,56^ In *C. necator*, the rhamnose-inducible promoter has been reported to offer even tighter regulation than the arabinose promoter,^37^ which is why we used it for lambda *red* expression. As demonstrated in this study, these strategies specifically designed for Beta, Exo and Gam production achieved controllable and highly efficient recombineering in *C. necator*.

We initially assessed the effectiveness of the lambda Red system by targeting an *eGFP* cassette inserted into the *phaC1* gene, which provided a robust fluorescence-based readout for recombination success. The results demonstrated that the Red system not only facilitated gene replacement through homologous recombination but also achieved a 40-fold increase in transformation rates compared to transformants not expressing lambda Red (Figure 2, C). While the enhanced transformation efficiencies initially surprised us, we realized that using low-stability plasmids as gene delivery vehicles allowed for immediate integration of the selection marker into the target locus, which appears advantageous under selection pressure. However, we also observed potential stability related problems associated with the used plasmid: The *appA* suicide plasmid could still be fully amplified by colony PCR days after electroporation into the lambda Red producing strain (Figure S5). Our suicide plasmids were intentionally designed with the origin of replication from pLO3. The pLO3 origin, a ColE-type origin derived from pBR322, is prevalent in Enterobacterales and other Gammaproteobacteria,^57^ but has been discussed as being incapable of autonomous replication in *C. necator*.^58^ ColE-type origins are also widely used in suicide plasmids for *C. necator* (18, 20, 41, 59). However, to our knowledge, these publications lack data on the half-life of these plasmids post-transformation. This fact and the observation that our own suicide plasmid does not disappear even after successful recombineering, raises concerns about whether plasmid curing is consistently and fully achieved. In contrast to suicide plasmids, efficient integration of linear DNA in all three cases (*eGFP*, *proC* and *eda*) was fully dependent on the recombineering system. Although transformation rates were lower, they were at least as effective as those achieved with the corresponding suicide plasmids. Linear vector DNA offers the advantage of being easily generated through overlap extension PCR, eliminating the need for *E. coli*-based plasmid assembly. Additionally, PCR products are not methylated, reducing the risk of restriction endonuclease-mediated electroporation issues.^18^ Since linear DNA fragments are typically degraded quickly by bacterial nucleases,^60−62^ they cannot be maintained in *C. necator* without genomic integration, which helps minimize the issues observed with suicide plasmids.

As proof of concept, we integrated a linear *appA* expression cassette via electroporation into the *phaC1* locus of wild type *C. necator* and the *eGFP* expression strain. The *appA* gene encodes for a bacterial phytase that breaks down phytate (IP6) into lower inositol phosphate forms (IP5-IP1) and inorganic phosphate.^63^ Since phytate cannot be utilized by monogastric animals but accounts for most plant derived phosphate, the addition of phytases to plant-based animal feeds increases the absorbable phosphate and is therefore considered an important feed supplementation.^64,65^ We recently published the production of AppA from highly stable episomal plasmids in *C. necator*.^21^ Although these plasmids are stably maintained in the expression host without any antibiotic selection pressure, they still carry the antibiotic resistance gene necessary for initial introduction. Especially in food and feed applications, the presence of an antibiotic resistance gene is undesirable due to concerns about a possible spread to pathogenic organisms. Furthermore, only a limited number of resistance markers are available for *C. necator* due to the organism’s high native resilience.^7^ To address this, we utilized the Cre/*loxP* site-specific recombinase system, the preferred method for removing genomically integrated marker cassettes.^59^ By applying a quick protocol for preparing competent cells, our marker recycling method became both fast and efficient, achieving success rates of 90–100%. The analysis of phytase activity in our marker-free production strain, which harbors only one copy of *appA*, was unexpectedly high, reaching 50% of the activity observed in an episomal system using a pBBR1-based plasmid, which likely contains 7–40 copies of the gene.^20,66^ Nile red staining and fluorescence microscopy of the *appA* expression strain confirmed the anticipated depletion of PHB due to the disruption of the *phaC1* locus. While PHB is of interest as a precursor for degradable bioplastics, it is indigestible for many higher eucaryotes and is therefore undesired in nutritional applications.^67−69^

Among the five lambda Red application examples presented in this study, editing efficiency varied substantially. While no clear correlation was found between deletion size and efficiency, larger insertions appeared to reduce integration frequency. This assumption is supported by a 60% decrease in editing efficiency when introducing the 1.7 kb *appA* cassette compared to the smaller knockout cassette (Figure S8). The differences in editing efficiency observed during the deletion of *eGFP*, *proC*, and *eda*, all using the same *Kan^R^* cassette, are likely influenced by gene-specific factors, such as the metabolic burden of the knockout, the sequence composition of the homologous arms, and the overall accessibility of the target locus.

One of the main advantages of our newly developed genome editing method is the short time required between gene delivery and obtaining the final engineered strains. With readily prepared competent cells that can be stored easily at −80 °C, it takes only 2 days to obtain the target strains. In contrast, using conjugational suicide plasmids for the same purpose typically takes twice as long due to the need for fresh cultures and the mating of *C. necator* with the donor *E. coli* S17-1 strain. This time frame does not account for the two rounds of restreaking sometimes recommended to avoid transconjugant contamination with the donor strain. Recently, Vajente et al.^70^ reported a combined approach using electroporation and a suicide plasmid containing a SacB/levan sucrose-based counterselection for marker-free gene deletions. Similar to our method, this approach overcomes the limitations of conjugation, and the transformation efficiency reported by Vajente et al. (8 cfu/μg DNA) is consistent with our observations using circular plasmids in wild-type *C. necator*. However, application of the lambda Red system resulted in approximately a 10-fold increase in transformation rates, and our Cre/*loxP* marker recycling strategy achieved a 100% success rate, which is significantly higher than the theoretical maximum of 50% achievable with the counterselection. Another method for electroporation-based genome engineering in *C. necator* is CRISPR/Cas9. However, the published method necessitates an additional 144–168 h induction period with arabinose to achieve editing efficiencies of 78–100%, which is comparable to the efficiency of our recombineering approach.^18^ Shorter induction times significantly reduce the observed editing efficiency for CRISPR/Cas9. Furthermore, the current electroporation-based CRISPR/Cas9 method only allows for gene deletions but still does not provide a working strategy for (marker-free) targeted insertion of recombinant DNA. Similarly, the RalsTron method, which utilizes group II introns for target-specific integration, only enables gene knockouts and exhibits a modest editing efficiency of 12.5%.^71^ In contrast, our lambda Red recombineering approach provides a quick and more versatile alternative. Furthermore, the licensing complexities associated with CRISPR/Cas9 make freedom-to-operate methods, such as the one described in this study, a more attractive choice.

In summary, our adapted lambda Red recombineering system offers a reliable and powerful tool for genome editing in *C. necator*. It enables efficient deletions and insertions, significantly reducing the time required for genome editing compared to the traditional conjugation methods and available CRISPR/Cas9 systems. The introduction of cassettes via electroporation further accelerates the process and minimizing the risk for genetic instability. Additionally, efficient marker recycling facilitates complex genome engineering, which is especially relevant in a bacterial host with significant potential in modern biotechnology.

## Materials and Methods

### Bacterial Strains and Cultivation Conditions

*E. coli* TOP10 (Invitrogen) was used for cloning and plasmid propagation. *C. necator* H16 (DSM 428) was the base strain for the knockout studies. Additionally, *C. necator* H16 PHB^–^4 (DSM 541) was used for the microscopy study.

*E. coli* was cultured at 37 °C in lysogeny broth (LB) medium, supplemented with either kanamycin [50 μg/mL], chloramphenicol [35 μg/mL] or tetracycline [12.5 μg/mL] depending on the antibiotic required for the respective plasmid. *C. necator* H16 was propagated at 28 °C and 170 rpm in tryptic soy broth (TSB) supplemented with either gentamicin [20 μg/mL], chloramphenicol [75 μg/mL], kanamycin [200 μg/mL] or tetracycline [10 μg/mL] depending on the application. For screening of *proC* and *eda* knockouts, *C. necator* H16 clones were selected on minimal medium plates, prepared as described by Lambauer et al.^7^ To facilitate growth of the corresponding strains, either 0.2% proline was supplemented or 2% lactate was used as carbon source instead of fructose. Agar was added to a final concentration of 2% for LB, TSB and minimal medium plates. All media components were purchased at CarlRoth.

### Cloning and DNA Delivery

Plasmids were constructed via Gibson Assembly^72^ and are listed together with used templates in Table 1. Detailed cloning strategies can be found in the Supporting Information. Plasmids were verified by restriction endonuclease digestion and Sanger sequencing. Linear fragments for electroporation were produced either by overlap extension PCR or by PCR amplification using suicide plasmids as templates, followed by agarose gel purification. All PCRs were performed using the Q5 High-Fidelity DNA Polymerase (New England Biolabs, Massachusetts, USA) following the manufacturer’s protocol. Homologous sequences for recombineering (ca. 900 bp) were initially amplified from genomic DNA of *C. necator*. For gel electrophoresis, the GeneRuler 1 kb Plus DNA ladder was used as a size standard. Detailed lists of all primers and a description of the target loci are provided in the Supporting Information (Tables S1–S11).

**Table 1:** Plasmids Used in This Work, Including a Description of Their Most Important Features, Their Application, and Source

Plasmid	Description	Application	Source
pCas	Kan^R^, P_BAD_, lambda Red genes (*gam*, *beta*, *exo*), pSC101 ori, Rep101, *SpyCas9*, gRNA	Template for amplification: *beta*, *exo*, *gam*	Addgene #62225, Jiang 2015^73^
pKRSF1010	Km^R^, RSF101 ori, RSF101 mob and T-ori	Template for amplification: Kan^R^	Gruber 2014^74^
pINT_lacY_Phac_loxP	*Cm*^R,^ pK470MobRP4, P_H16 B1772_, *lacY*, *phaC1* homologous regions, *loxP* sites	Vector backbone used for *eGFP* integration	Gruber 2016^56^
pBR322	Tet^R^, Amp^R^, pMB1 ori, *rop*	Template for amplification: tetR	NEB #N3033S, Bolivar 1977^75^
pKESa	Km^R^ P_t5_, *eGFP*, pSa ori	Template for amplification: pSa	Arhar 2024^21^
pKPRepPar_Pj5	Km^R^, P_j5_, *E. coli appA*, pBBR1 ori, *par*	Template for amplification: *appA*	Arhar 2024^21^
pLO3	Tet^R^, *sacB*, RP4 transfer ori, pBR322 ori	Template for amplification: ori pLO3	Lenz 1998^41^
pCM_Cre	*Cm*^R^, P_tac-CyO_, mob, colE1 ori, *cre*, *cym*^*R*^	Template for amplification: *Cm*^R^; P_Tac-CyO_; *cre*; *cym*^*R*^	Gruber 2016^59^
pINT_eGFP_Phac_loxP	*Cm*^R,^ pK470MobRP4, P_t5_, *eGFP*, *phaC1* homologous regions, *loxP* sites	Integration of an *eGFP* expression cassette into *phaC1*	This study
pCn-Red	Tet^R^, pSa ori, P_rha_, *beta*, *exo*, *gam*,, *rhaS*, *rhaR*	Induced expression of the lambda *red* system in *C. necator*	This study
pHRep-HKH1	Km^R^, pLO3 ori, *phaC1* homologous regions	Replacement of *eGFP*/disruption of *phaC1*, vector backbone for pHInt-AppA-KanR-loxP	This study
pHRep-HKH-ProC	Km^R^, pLO3 ori, *proC* homologous regions	Replacement of *proC*	This study
pHRep-HKH-Eda	Km^R^, pLO3 ori, *eda* homologous regions	Replacement of *eda*	This study
pHInt-AppA-KanR-loxP	Km^R^, pLO3 ori, *loxP* sites, *phaC1* homologous regions, P_j5_*appA*	Disruption of *phaC1* via integration of *appA*	This study
pCn-Cre	*Cm*^R^, pLO3 ori, *cre*, *cym*^*R*^, P_Tac-CyO_	Production of *C*re recombinase for marker recycling	This study

Electro-competent *C. necator* cells were prepared following the protocol from Taghavi et al. with slight modifications.^17,21^ Briefly, a single colony was used to inoculate 3 mL of SOB medium (5 g/L yeast extract, 20 g/L tryptone, 0.6 g/L NaCl, 0.2 g/L KCl) for overnight cultivation at 28 °C. The next day, a main culture of 200 mL SOB in 1 L unbaffled shake flasks was inoculated to a start-OD_600_ of 0.05 and incubated at 28 °C and 170 rpm. Once an OD_600_ of 0.6–0.8 was reached, the culture was chilled on ice for 30 min and split into four. The cells were harvested by centrifugation at 3 200 rcf for 10 min at 4 °C. Each pellet was resuspended in 50 mL of 15% (w/v) glycerol and centrifuged at 3 200 rcf for 10 min at 4 °C. The washing step was repeated with 30 and 5 mL of 15% (w/v) glycerol. Finally, for each 0.1 OD_600_ unit initially harvested, 75 μL of 15% (w/v) glycerol were added to the pooled final pellet. Suspended aliquots of 50 μL were shock-frozen in liquid nitrogen and stored at −80 °C.

Electroporation of *C. necator* H16 was performed using a MicroPulser electroporator (Bio-Rad, USA) with prechilled 2 mm electroporation cuvettes at 2.5 kV/cm. Plasmids (300 ng) and linear DNA fragments (1000–1500 ng) were used for transformation. Cells were allowed to recover in 1 mL of SOC medium (20 g/L tryptone, 5 g/L yeast extract, 0.58 g/L NaCl, 2 g/L MgCl_2_·6H_2_O, 0.186 g/L KCl, 2.46 g/L MgSO_4_·7H_2_O, 3.96 g/L glucose-monohydrate) at 28 °C for 2 h before being plated on TSB agar containing the appropriate antibiotic. The plates were incubated at 28 °C for 2–3 days.

During preparation of the competent recombineering cells, *C. necator* carrying the pCn-Red plasmid was cultivated in the presence of tetracycline. To induce expression of lambda *exo*, *beta* and *gam*, 10 mM rhamnose was added to the SOB media at an OD_600_ of 0.3–0.4. After one last doubling, cells were harvested and treated as previously described for preparation of competent cells and electroporation. Selection for vector integration was done by only adding kanamycin to TSB agar plates.

Electroporations for each target vector were performed in biological triplicates, to determine accurate transformation rates and editing efficiencies. Transformants were initially screened based on the phenotype, as detailed in the Results section. Editing efficiencies were calculated as the ratio of transformants showing the appropriate phenotype per total number screened (50 clones if electroporation rates sufficed). Following phenotypic evaluation, genotypic validation was performed via colony PCR with primers designed to differentiate correct mutants from wild-type by amplicon size. Template DNA was prepared by suspension of small amounts of colonies into 25 μL of water, heating for 5 min to 95 °C, cooling on ice for additional 5 min and sedimentation of cell debris via centrifugation. For a 10 μL PCR 0.5 μL of the prepared supernatant were used. For each transformation, 10 clones exhibiting the expected phenotype were selected for genotypical verification.

### Strain Construction

The *C. necator* strain for genomic *eGFP* expression was created by modifying the previously published pInt_lacY_phaC_loxP plasmid.^59^ The original *lacY* was replaced with an *eGFP* expression cassette controlled by a constitutive t5 promotor. Conjugation, using the *E. coli* S17-1 strain as a donor, was employed to introduce the plasmid into *C. necator* H16. Overnight cultures of *E. coli* S17-1 carrying the *eGFP* expression plasmid and *C. necator* H16 were diluted back to an optical density of 0.1 and 0.3, respectively. After 4 h of incubation, five and seven OD_600_-units of each culture were harvested, suspended in 600 μL of 0.9% sodium chloride solution, and combined. After centrifugation at 16 000 rcf for 30 s, the pellet consisting of *E. coli* S17-1 and *C. necator* H16 cells was suspended in 100 μL sodium chloride solution, spotted on a TSB plate without antibiotics, and incubated overnight. After incubation, the spots were scraped off, suspended in 1 mL of 0.9% sodium chloride and plated in appropriate dilutions (typically undiluted-10^–3^) on TSB agar. Selection for genomic integration of the *eGFP* expression cassette into the *phaC1* locus was achieved with 100 μg/mL chloramphenicol, while 20 μg/mL gentamicin was used to eliminate *E. coli* contaminations. Colonies appearing after 2 days of incubation were restreaked twice to obtain pure *C. necator* strains. Correct integration was confirmed by colony PCR with primers specific for the genomic locus and Sanger sequencing.

### Minimal Transformation Protocol for *C. necator*

For iterative transformations of *C. necator*, a streamlined protocol for generating electrocompetent *E. coli* cells was adapted by some minor modifications.^44^ SOB medium (3 mL) supplemented with antibiotic was inoculated with a single colony and incubated overnight at 28 °C. The following day, a main culture of 20 mL SOB medium was inoculated to an OD_600_ of 0.1 and grown at 28 °C until an OD_600_ of 0.6 was reached. The cultures were then harvested by centrifugation at 3 200 rcf for 5 min at 4 °C. The cell pellets were washed twice with 20 mL ice cold 0.2 M sucrose and after a final centrifugation, suspended in 50 μL ice-cold 0.2 M sucrose.^76^ The cells were ready for immediate transformation using the described electroporation protocol.

### Phytase Activity Assay

For the enzymatic phytate hydrolysis reactions, cell free extracts of *C. necator* cells were prepared using the standard BugBuster protocol (Novagen, Merck), with the reagent diluted 1:10 in Tris/HCl buffer (100 mM, pH 8). As previously discribed,^21^ the cell-free extracts were diluted 1:2 in reaction buffer (100 mM acetate buffer, pH 4.5) and mixed with a sodium phytate stock (10x in water) to reach a final substrate concentration of 8 mM in 0.5 mL reaction volume. Reactions were performed at 37 °C with gentle shaking. After 0 and 15 min, 125 μL samples were taken, deactivated by adding 25 μL of 3 M HCl and analyzed for their phosphate concentration using the Saheki method.^77^ Therefore, 10 μL of each deactivated sample were transferred to a 96-well plate and 120 μL Saheki solution (12 mM ammonium molybdate, 80 mM zinc acetate and 2% ascorbic acid, pH 5) was added. After 15 min at room temperature, the samples were measured at 850 nm using the Eon High Performance Microplate Spectrophotometer (BioTek Instruments, Inc.). KH_2_PO_4_ standards were used for calibration. All measurements were performed in biological triplicates and technical duplicates. To avoid high background levels of phosphate, highly pure water (max 18.2 MΩ cm) was used for the preparation of all solutions applied in the assay.

### PHB Staining

The Nile red staining of *C. necator* was performed following the protocol of Li et al.^78^ with minor modifications. Precultures of 3 mL minimal medium with 2% fructose were inoculated with fresh colonies and shaken at 28 °C for 24 h. Main cultures containing the same media were inoculated to an OD_600_ of 0.3 and shaken at 28 °C for 48 h. 200 μL of each culture were harvested by centrifugation at 1 500°rcf for 5 min. The pellets were washed in 400 μL of 0.9% sodium chloride and the pellets were resuspended in 200 μL of 0.9% sodium chloride. For PHA staining, 4 μL of Nile red (1 500 μg/mL in DMSO, Sigma-Aldrich) were added. The cells were incubated in the dark for 30 min, then washed again with 400 μL 0.9% sodium chloride.

For microscopy, agarose slides (1%) were prepared to ensure proper mounting of the cells. Microscopic analysis of the strains was performed on a Zeiss Axio Imager.M2 using the EC Plan-Neofluar 100×/1.3 Oil M27 objective and LED module 567 as light source. For visualization of Nile red stained PHA granule, excitation and emission wavelength were adjusted to 574–599 nm and 612–682 nm via optical filters, respectively. Whole cells were imaged in bright field mode.

### Plasmid Loss and Marker Recycling

Recycling of the kanamycin resistance cassette was done by bordering *loxP* sites and a Cre-recombinase supplied on the pCn-Cre plasmid. The plasmid was introduced into *C. necator* via the minimal electroporation protocol. Cells were plated on TSB agar containing chloramphenicol (75 μg/mL) and 120 mM p-cumate. The transformants received after 2 days of incubation at 28 °C already lost kanamycin resistance.

To ensure the loss of the pCn-Red plasmid, a single colony of *C. necator* cells harboring pCn-Red was inoculated into 3 mL of minimal medium with gentamycin (culture 1) and incubated at 28 °C with shaking for 48 h. Then, 50 μL of culture 1 were used to inoculate a second 3 mL minimal medium culture with gentamycin (culture 2). Culture 2 was again incubated at 28 °C with shaking for 48 h. The OD_600_ of culture 2 was measured, and an appropriate dilution of the culture was plated on TSB agar with gentamycin to obtain approximately 100 colonies. After incubation at 28 °C for 24 h, the resulting colonies were restreaked on TSB agar with and without tetracycline to identify clones that have lost the pCn-Red plasmid.

## Supporting Information Available

Detailed cloning strategies and sequences of all used primers (Tables S1–S12); additional data related to the preparation of competent cells with different recombineering systems (Figures S1 and S2), the optimization of used DNA amounts for recombineering (Figure S3), the marker-free integration on an *appA*-expression cassette into *C. necator* (Figures S4–S7), and a summary of editing efficiencies achieved during the study (Figure S8); sequences of the plasmids created during this study (PDF)
