# Supplementary material for: CnRed: Efficient, Marker-free Genome Engineering of Cupriavidus necator H16 by Adapted Lambda Red Recombineering
Source: ACS Synth Biol. 2025 Feb 24;14(3):842–54. doi: 10.1021/acssynbio.4c00757 (PMC11934132; doi:10.1021/acssynbio.4c00757)
Supplement: Supplementary file 1 — sb4c00757_si_001.pdf [file sb4c00757_si_001.pdf]

'CnRed: Efficient, marker-free genome engineering of *Cupriavidus necator* H16 by adapted Lambda Red recombineering.'

Simon Arhar[1,3†], Johanna Pirchner[1†], Holly Stolterfoht-Stock[1], Karin Reicher[1], Robert Kourist[1,2], Anita Emmerstorfer-Augustin[1,2,3*].

[1] Austrian Centre of Industrial Biotechnology, acib GmbH, 8010 Graz, Austria
[2] Institute of Molecular Biotechnology, Graz University of Technology, NAWI Graz, 8010 Graz, Austria
[3] BioTechMed-Graz, 8010 Graz, Austria

† These authors contributed equally to this work.

*Corresponding Author:

DI Dr. Anita Emmerstorfer-Augustin

Austrian Center of Industrial Biotechnology

Krenngasse 37

8010 Graz

emmerstorfer-augustin@tugraz.at

Tel.: +43 316 873 4078

Supplementary Information

1. Supporting information

The supporting information contains a detailed description of the three targeted *C. necator* loci (Table S1) and detailed descriptions of cloning strategies (Table S2 – S12). Further, additional data concerning optimization of the recombineering (Figure S1-S3), the *appA* integration studies (Figure S4 – S7) and a summary of editing efficiencies achieved during the study (Figure S8) are provided.

1.1 Loci targeted in this work

Table S1: *Cupriavidus necator* loci information, including their RefSeq, location, locus tag and chromosome number.

Gene	RefSeq-Protein	Location	Locus tag	Chromosome Nr.
proC	WP_011615991.1	3359051…3359887	H16_A3106	1
eda	WP_010814144.1	1370637…1371281	H16_B1213	2
phaC1	WP_013956451.1	1556003…1557772	H16_A1437	1

1.2 Cloning of plasmids used in this study

The fragments *phaC1 5', phaC1 3', proC 5', proC 3', eda 5'* and *eda 3'* were amplified from the genomic DNA (gDNA) of *C. necator* H16 wild-type.

Plasmid pINT_eGFP_PhaC_loxP

To construct a conjugational suicide plasmid for integrating an *eGFP* cassette into the *phaC1* locus, we utilized the previously published plasmid pINT_lacY_phaC-loxP (Table 1). The plasmid was digested with *Sph*I and *Hind*III to excise the *lacY* expression cassette. The *eGFP* cassette, which includes the t5 promoter and the target gene, was amplified from pKESa using primers pCR-Int-eGFP_f1/r1. Gibson assembly was then performed with the digested backbone and the PCR product. The final construct was verified by restriction digest and Sanger sequencing, using primers pSEQ-pInt-phaC_f1 and pSEQ-pInt-CmR_r1. To confirm correct genomic integration, colony PCR was conducted with primers pCont_phaC1_f2/r2, followed by sequencing of the corresponding DNA fragment. For the primer sequences, please refer to Table S2.

Table S2: Primers used for construction and further experiments related to pINT_eGFP_PhaC_loxP. Capital letters refer to the primer part binding to the target sequence.

Application	Primer name	Sequence
PCR: P_{t5}-eGFP	pCR-Int-eGFP_f1	gcagcacctgggacgactactagtgcatgcAAATCATAAAAAATTTATTTGCTTTGTGAGCG
	pCR-Int-eGFP_r1	cttctctcatccgccaaaacagccaagcttTTACTTGTACAGCTCGTCCATGC
Colony PCR	pCont_phaC1_f2	GGAGCCGGTTCGAATAGTGAC
	pCont_phaC1_r2	GACAACGTCAGTCATTGTGTAGTC
Sequencing	pSEQ-pInt-phaC_f1	GTACTACATCCTGGACCTGCAG
	pSEQ-pInt-CmR_r1	CTTCCGTCACAGGTATTTATTCGTC

Plasmid pCn-Red

The recombineering plasmid was constructed by Gibson Assembly of the following PCR products. The pSa *ori* was amplified from the plasmid pKESa using primers pCR-pCn-RED-SaOri_f1/r1. The tetracycline resistance cassette was amplified from the plasmid pBR322 (Table 1) using primers pCR-pCn-RED-TetR_f1/r1. Promoter P_{rha} with regulators *rhaR/S* was amplified from genomic DNA of *E. coli* MG1655 (Table 1) using primer pCR-Reco-rha_f/f1/r1. *Beta*, *exo* and *gam* were amplified from plasmid pCas (Table 1) using primer pCR-Beta_f1/r1, pCR-Exo_f1/r1 and pCR-Gam_f1/r1, respectively. *Cuprividus necator recA* was amplified using primer pCR-CnRecA_f1/r1 and genomic DNA of strain H16 as template. The genes *recE* and *recT* were amplified form genomic DNA of *E. coli* MG1655 (Table 1) using primers pCR-EcRecE_f1/r1 and pCR-EcRecT_f1/r1, respectively. Correct assembly was confirmed by Sanger sequencing using the primer pSeq-pSa-Rep_f1b, pSeq-rhaR_r1, pSeq-rhaS_r1, pSeq-PrhaB_r1, pSeq-TetR_r1, pSeq-recT_f1, pSeq-recE_f1 and pSeq-recE_f2. Sequences of all primers mentioned above can be found in Table S3.

Table S3: Primers used to construct plasmid based recombineering systems. Capital letters indicate the primer regions that bind to the target sequence.

**Application**	**Primer name**	**Sequence**
PCR: TetR	pCR-pCn-RED-TetR_f1	CCGAAAAGTGCCACCTGACG
	pCR-pCn-RED-TetR_r1	agtttgagatctttaattaatttatgtgatatttaaatTGGAGTGGTGAATCCGTTAGC
PCR: pSa	pCR-pCn-RED-SaOri_f1	taaatatcacataaattaattaaagatctcaaactgtacgtttaaacGCTACTTTCCGAACGACTCCTG
	pCR-pCn-RED-SaOri_r1	cctgcagGGCAGACCAGAACCAATCCTATTC
PCR: P_{rHA} + rhaR + rhaS	pCR-Reco-rha_f	cgcagaaaggcccacccgaaggtgagccagtgtgactctagtagtttaaacttaatctttctgcgAATTGAGATGACGC
	pCR-Reco-rha_f1	ccttcgtttaggtgctgaataggattggttctggtctgccctgcaggTCTAGAcgcagaaaggcccacc
	pCR-Reco-rha_r1	atgtatatctccttcttaagaattgttcatTACGACCAGTCTAAAAAGCg
PCR: *beta*	pCR-Beta_f1	actggtcgtaatgaacaattcttaagaaggagatatacatATGAGTACTGCACTCGCAAC
	pCR-Beta_r1	catcgatcccggtacgctgcaggataatgtccggtgtcatggttgtcctcctttTCATGCTGCCACCTTCTG
PCR: *exo*	pCR-Exo_f1	ATGACACCGGACATTATCCTG
	pCR-Exo_r1	aatgcttttgcttgatctcagtttcagtattaatatccatggttgtcctcctttTCATCGCCATTGCTCCC
PCR: *gam*	pCR-Gam_f1	ATGGATATTAATACTGAAACTGAGATCAAGC
	pCR-Gam_r1	aatggtttcttagacgtcaggtggcacttttcggaaaaggccatccgtcaggatggccttctTTATACCTCTGAATCAATATCAACCTGG
PCR: *recA*	pCR-CnRecA_f1	actggtcgtaatgaacaattcttaagaaggagatatacatATGGACGACAAGAAGGCAGG
	pCR-CnRecA_r1	taatggtttcttagacgtcaggtggcacttttcggaaaaggccatccgtcaggatggccttctGGCGCGCC TCAGTCTTCGACCGCTACC
PCR: *recT*	pCR-EcRecT_f1	actggtcgtaatgaacaattcttaagaaggagatatacat ATGACTAAGCAACCACCAATCG
	pCR-EcRecT_r1	ttttgtgctcatggttgtcctcctttt TTATTCCTCTGAATTATCGATTACACTGTATTCC
PCR: *recE*	pCR-EcRecE_f1	attcagaggaataaaaaggaggacaacc ATGAGCACAAAACCACTCTTCC
	pCR-EcRecE_r1	ataatggtttcttagacgtcaggtggcacttttcggaaaaggccatccgtcaggatggccttct TTAGTCATTTGCATATTCCTTAGCCC
Sequencing	pSeq-pSa-Rep_f1b	GAATTAAATACCCTGTTGCGGTATAG
	pSeq-rhaR_r1	CAGTCAAGATTCAGCTTCAGACG
	pSeq-rhaS_r1	CTGCTGTTCCATCTGTGCAACC
	pSeq-PrhaB_r1	CAAATTGTGAACATCATCACGTTCATC
	pSeq-TetR_r1	CTTATCGATGATAAGCTGTCAAACATG
	pSeq-recT_f1	ACGGCTATTCGTCGCCTGTTC
	pSeq-recE_f1	CGAAGCATACCTGAACGCTGAC
	pSeq-recE_f2	GGAATTAGCGACCCGAACGATC

Suicide plasmid pHRep-HKH1

The suicide plasmid pHRep-HKH1 was constructed using Gibson Assembly. The origin of replication was amplified from the plasmid pLO3 (Table 1) with primers pCR-HKH1-ColE1_f1/r1. The kanamycin resistance cassette was amplified from the plasmid pKRSF1010 (Table 1) using primers pCR-HKH1-KanR_f1/r1. The 5' and 3' homologous regions of *phaC1* were amplified from the genomic DNA of *C. necator* H16 wild-type (H16_A1437; see Table S1) using primers pCR-HKH1-phaC1-1_f1/r1 and pCR-HKH1-phaC1-2_f1/r1, respectively. Correct assembly was confirmed by Sanger sequencing using primers pSeq-KanR_f1, pSEQ-pInt-phaC_f1, pCont_phaC1_r1, pSeq-KanR_r1, pCont_BB_f1, and pSeq-phaC1_2_f1. After transformation into *C. necator*, the replacement of the genomic *eGFP* was verified via colony PCR using primers pCont_phaC1_f2/r2 (Table S2). The sequences of the primers used for the construction and sequencing of pHRep-HKH1 can be found in Table S4.

Table S4: Primers used for construction of pHRep-HKH1 and the following experiment with the plasmid. Capital letters refer to the primer part binding to the target sequence.

Application	Primer name	Sequence
PCR: ori pLO3	pCR-HKH1-ColE1_f1	aagccaaggcatgactcgagATGTGAGCAAAAGGCCAGCAAAAG
	pCR-HKH1-ColE1_r1	ggtcgccattctagaCCCGTAGAAAAGATCAAAGGATCTTCTTGAG
PCR: Kan^R	pCR-HKH1-KanR_f1	tgggacgactgaattcTTTGCGTTTCTACAAACTCTTTTGTTTATTTTTC
	pCR-HKH1-KanR_r1	gccgcgtgctcgatgtggatccAAGGCCATCCGTCCTAGGAG
PCR: phaC1_1	pCR-HKH1-phaC1-1_f1	aagatcctttgatcttttctacgggtctagaATGGCGACCGGCAAAGGCG
	pCR-HKH1-phaC1-1_r1	caaaagagtttgtagaaacgcaaagaattcAGTCGTCCCAGGTGCTGC
PCR: phaC1_2	pCR-HKH1-phaC1-2_f1	cctaggacggatggccttggatccACATCGAGCACGCGGCCATC
	pCR-HKH1-phaC1-2_r1	tgctggccttttgctcacatctcgagTCATGCCTTGGCTTTGACGTATCG
Sequencing	pSeq-KanR_f1	GCCTCGGTGAGTTTTCTCCTTC
	pSEQ-pInt-phaC_f1	gtactacatcctggacctgcag
	pCont_phaC1_r1	CGCGGTCGAGACAATGGTG
	pSeq-KanR_r1	CTAGAGCAAGACGTTTCCCGTTG
	pCont_BB_f1	CCA CCT CTG ACT TGA GCG TCG
	pSeq-phaC1_2_f1	atcgagcatcacggcagctg

Linear fragment PCR-HKH1

The linear fragment PCR-HKH1 was amplified using the suicide plasmid pHRep-HKH1 as a template with the primers pInt-phaC1-eGFPlin_f1/r1 (Table S5). After transformation into *C. necator* (procedure outlined in Materials and Methods section 4.2), the replacement of genomic *eGFP* was determined via colony PCR using the primer pCont_phaC1_f2/r2 (Table S2).

Table S5: Primers for the amplification of PCR-HKH1. Capital letters refer to the primer part binding to the target sequence.

Application	Primer name	Sequence
PCR: amplification of linear fragment	pInt-phaC1-eGFPlin_f1	ATGGCGACCGGCAAAGGCG
	pInt-phaC1-eGFPlin_r1	TCATGCCTTGGCTTTGACGTATCG
PCR: amplification of linear fragment (60 bp HR)	pCR-recombK_f1	tgtttctggtgtcgtggcgcaatccggacgccagcatggccggcagcacctgggacgactTTCTTTGCGTTTCTACAAACTCTTTTG
	pCR-recombK_r1	cgagacaatggtgccgcccacgcagaagccgagcacgttgatcttgtcctggccgctgatCCTGTCTGACGCTCAGTGG

Suicide plasmid pHRep-HKH-ProC

For the construction of the suicide plasmid pHRep-HKH-ProC, the origin of replication from pLO3 and the kanamycin resistance cassette from plasmid pHRep-HKH1 were amplified using primers pCR-ori_pLO3_f2/r2 and pCR-Hrep-Kan_f2/r2, respectively. The 5' and 3' homologous regions of *proC* were amplified from the genomic DNA of *C. necator* H16 wild-type (Table S1) using primers pCR-HRep-proC-5'_f1/r1 and pCR-HRep-proC-3‘_f1/r1. Correct assembly was confirmed by Sanger sequencing using six different primers. After transformation into *C. necator*, the replacement of the genomic *proC* was verified via colony PCR using primers pCont_proC_f1 and pSeq-KanR_r1. The sequences of the primers used are summarized in Table S6.

Table S6: Primers used to construct pHRep-HKH-ProC and analyze target recombination events. Capital letters indicate the primer regions that bind to the target sequence.

Application	Primer name	Sequence
PCR: ori pLO3	pCR-ori_pLO3_f2	ctcgagATGTGAGCAAAAGGCCAGCAAAAG
	pCR-ori_pLO3_r2	tctagaCCCGTAGAAAAGATCAAAGGATCTTCTTGAG
PCR: Kan^R	pCR-Hrep-Kan_f2	gaattcTTTGCGTTTCTACAAACTCTTTTGTTTATTTTTC
	pCR-Hrep-Kan_r2	agatctAAGGCCATCCGTCCTAGGAG
PCR: ProC 5'	pCR-HRep-proC-5‘_f1	tctcaagaagatcctttgatcttttctacgggtctagaACATCCTTGTGCGTCATCGAGG
	pCR-HRep-proC-5‘_r1	aaaataaacaaaagagtttgtagaaacgcaaagaattcATCGAGCATGGAGATCCGTTGG
PCR: ProC 3'	pCR-HRep-proC-3‘_f1	tgagcgtcagacaggcctcctaggacggatggccttagatctATTGAGGCGCGGCCAAAC
	pCR-HRep-proC-3‘_r1	tacggttcctggccttttgctggccttttgctcacatctcgagGCACGCTGCTGCTGCTG
Colony PCR	pCont-proC_f1	TGTCGCTCATGATGTCGTACAC
	pSeq-KanR_r1	CTAGAGCAAGACGTTTCCCGTTG
Sequencing	pSeq-KanR_r1	CTAGAGCAAGACGTTTCCCGTTG
	pSeq-ori-pLO3_f1	AAAGAGTTGGTAGCTCTTGATCCG
	pSeq-KanR_f1	GCCTCGGTGAGTTTTCTCCTTC
	pCont_BB_f1	CCACCTCTGACTTGAGCGTCG
	pCR-Hrep-Kan_f2	GAATTCTTTGCGTTTCTACAAACTCTTTTGTTTATTTTTC
	pCR-ori_pLO3_f2	CTCGAGATGTGAGCAAAAGGCCAGCAAAAG

Linear fragment PCR-HKH-ProC

The amplification of the kanamycin resistance cassette and of the elements *proC 5'* and *proC 3'* is outlined in Table S6. These are the same elements used for the construction of the suicide plasmid pHRep-HKH-ProC. The fragment for transformation into *C. necator* was generated via overlap extension PCR using the primers pRec-ProC_f2/r2 (Table S7). After transformation, the replacement of genomic *proC* was determined via colony PCR using the primer pCont_proC_f1 and pSeq-KanR_r1 (Table S6).

Table S7: Primers used for amplification PCR-HKH-ProC.

Application	Primer name	Sequence
Overlap extension PCR	pRec-ProC1_f2	ACATCCTTGTGCGTCATCGAGG
	pRec-ProC1_r2	GCACGCTGCTGCTGCTG

Suicide plasmid pHRep-HKH-Eda

For the construction of suicide plasmid pHRep-HKH-Eda, the pLO3 origin of replication and kan^R for the construction of plasmid pHRep-HKH1 were reused (primer for *ori* and kan^R see Table S6). *Eda 5'* and *eda 3'* homologous regions were amplified from the gDNA of *C. necator* H16 wildtype (Table S1) using primer pCR-HRep-eda-5'_fw/rv and pCR-HRep-eda-3'_fw/rv, respectively (Table S8). Correct assembly was confirmed by Sanger sequencing using the same six primers as for the *proC* plasmid (Table S6). After transformation into *C. necator*, the replacement of the genomic *eda* locus was verified via colony PCR using the primer pCont-eda_f1 and pCR-Rep-eda-3in1_r1 (Table S8).

Table S8: Primers used to construct pHRep-HKH-Eda and verify correct recombination into the *eda* locus. Capital letters indicate the primer regions that bind to the target sequence.

Application	Primer name	Sequence
PCR: Eda 5'	pCR-HRep-eda-5'_f1	GGATCTCAAGAAGATCCTTTGATCTTTTCTACGGGTCTAGACATCGCGCTACCTGCAAGG
	pCR-HRep-eda-5'_r1	aaaaataaacaaaagagtttgtagaaacgcaaaGAATTC TCAGGGAAGCAGGAGTTCAGG
PCR: Eda 3'	pCR-HRep-eda-3'_f1	gagcgtcagacaggcctcctaggacggatggccttAGATCT GAGAAACGGCACGCCATGC
	pCR-HRep-eda-3'_r1	CGGTTCCTGGCCTTTTGCTGGCCTTTTGCTCACATCTCGAGCCGCTGCAATTCACCCTGC
Colony PCR	pCont-eda_f1	GAACCGCTGGTGGAGTTCAAC
	pCR-Rep-eda-3in1_r1	CATGGCGTGCCGTTTCTC

Linear fragment PCR-HKH-Eda

Amplification of the kanamycin resistance cassette is outlined in Table S6, amplification of the homologous regions *eda 5'* and *eda 3'* is outlined in Table S8. These are the same fragments present on the respective suicide plasmid pHRep-HKH-Eda. The final linear fragment was assembled by overlap extension PCR using the primer pRec-eda-fw1/rv1 (Table S9). After transformation into *C. necator*, the replacement of the genomic *eda* was determined via colony PCR using the primer pCont-eda_f1 and pCR-Rep-eda-3in1_r1 (Table S8).

Table S9: Primers for production of PCR-HKH-Eda.

Application	Primer name	Sequence
Overlap extension PCR	pRec-eda-f1	CATCGCGCTACCTGCAAGG
	pRec-eda-r1	CCGCTGCAATTCACCCTGC

Suicide plasmid pHInt-AppA-KanR-loxP

The suicide plasmid pHInt-AppA-kanR-loxP was constructed using Gibson Assembly. The plasmid pHRep-HKH1 was used as backbone with *kan*^{R} being excised using restriction enzymes *Eco*RI and *Bam*HI. The fragment *appA* including one *loxP* site was amplified from the plasmid pKPRepPar_Pj5 (Table 1) using primer pCR-AppA-loxP-PhaC_f1 and pCR-AppA-loxP_r1 with the *loxP* site being added as primer overhang. Kan^R cassette including the second *loxP* site was amplified from the plasmid pHRep-HKH1 with *loxP* using primer pCR-KanR-loxP-PhaC_f1 and primer pCR-KanR-loxP_r1 with again the *loxP* site being added as primer overhang. Correct assembly was confirmed by Sanger sequencing using primer pSeq-ori-pLO3_f1, pSeq-KanR_r1, pSeq-rrnbTT_r1 and pSeq-rrnbTT_f1. After transformation into *C. necator*, the replacement of the genomic *phaC1* locus was determined via colony PCR using the primer pCont-PhaC1_f2/r2 (Table S2). The sequences of the additional primers used during construction of pHInt-AppA-KanR-loxP can be found in Table S10.

Table S10: Primers used during the construction of plasmid pHInt-AppA-KanR-loxP and for analysis of correct genomic integration. Capital letters indicate the primer regions that bind to the target sequence.

Application	Primer name	Sequence
PCR: AppA + loxP	pCR-AppA-loxP-PhaC_f1	gacgccagcatggccggcagcacctgggacgactGCATGCaaaaaccgttattgacacag
	pCR-AppA-loxP_r1	aaacaaaagagtttgtagaaacgcaaaCATATGataacttcgtatagcatacattatacgaagttatTTAATTAAaaggccatccgtcag
PCR: Kan^R + loxP	pCR-KanR-loxP-PhaC_f1	CATATGtttgcgtttctacaaactcttttgttt
	pCR-KanR-loxP_r1	cttcgatggcgcggatggccgcgtgctcgatgtataacttcgtatagcatacattatacgaagttatACTAGTaaggccatccgtcctag
Colony PCR	pCont-PhaC1_f4	CTTCCAGCCAGTTCCAGG
	pCont-PhaC1_r4	GACTTCGCTCACCTGCTC
	pCont_BB_f1	CCACCTCTGACTTGAGCGTCG
Sequencing	pSeq-ori-pLO3_f1	AAAGAGTTGGTAGCTCTTGATCCG
	pSeq-KanR_r1	CTAGAGCAAGACGTTTCCCGTTG
	pSeq-rrnbTT_r1	cgtttcacttctgagttcggcatg
	pSeq-rrnbTT_f1	gcggatttgaacgttgcgaagc

Linear fragment pHInt-AppA-KanR-loxP

The linear fragment pHInt-AppA-KanR-loxP was amplified from the respective suicide plasmid pHInt-AppA-KanR-loxP using primer pInt-phaC1-eGFPlin_f1/r1 (Table S5). After transformation into *C. necator* the replacement of genomic *phaC1* and *eGFP,* respectively was determined via colony PCR using the primer pCont-phaC1_f4/r4 (Table S11).

Table S11: Primers for analysis of the target locus for *appA* integration.

Application	Primer name	Sequence
Colony PCR	pCont-PhaC1_f4	CTTCCAGCCAGTTCCAGG
	pCont-PhaC1_r4	GACTTCGCTCACCTGCTC

Plasmid pCn-Cre

The plasmid pCn-Cre was constructed using Gibson Assembly. The fragment comprising P_{tac}, the Cre recombinase, *Cm^R^* and cymR were amplified from the plasmid pCM_Cre (Table 1) using primers pCR-CymR-pLO3_f1 and pCR-pCnIC-CmR_r1, respectively. The pLO3 origin of replication was amplified from plasmid pLO3 (Table 1) using the primers pCR-pCnIC-ori_f1 and pCR-pCnIK-ori_r1. Correct assembly was confirmed by Sanger sequencing using primer pSeq-ori-pLO3_f1 and pSEQ-pInt-CmR_r1. Successful removal of the kanamycin resistance cassette in strains of interest was analyzed via colony PCR using primer pCont_phaC1_f1/r1. Sequences of all primers utilized during the construction of pCn-Cre and marker recycling can be found in Table S12.

Table S12: Primer used to construct plasmid pCn-Cre and for investigation of marker recycling. Capital letters indicate the primer regions that bind to the target sequence.

Application	Primer name	Sequence
PCR: P_{tac} + cre + Cm^R + cym^R	pCR-CymR-pLO3_f1	AAGGATCTCAAGAAGATCCTTTGATCTTTTCTACGGGGTCGAC acggatggcctttttgc
	pCR-pCnIC-CmR_r1	CCTGGCCTTTTGCTGGCCTTTTGCTCACATttaactggcctcaggcatttg
PCR: ori pLO3	pCR-pCnIC-ori_f1	ATGTGAGCAAAAGGCCAGC
	pCR-pCnIK-ori_r1	CCCGTAGAAAAGATCAAAGGATCTTC
Sequencing	pSeq-ori-pLO3_f1	AAAGAGTTGGTAGCTCTTGATCCG
	pSEQ-pInt-CmR_r1	CTTCCGTCACAGGTATTTATTCGTC
Colony PCR	pCont_phaC1_f1	TCTTCGAGAACGAGTACTTCCAG
	pCont_phaC1_r1	CGCGGTCGAGACAATGGTG

1.3 Additional data

Three distinct approaches were initially tested to enhance recombination efficiency in *C. necator*: the polycistronic overexpression of the recombineering systems lambda *red* and *recET*, as well as the overexpression of *C. necator recA* from the rhamnose-inducible promoter. For each of these potential recombineering systems, two induction durations were evaluated, corresponding to either one or three cell density doublings. During the cultivation of *C. necator* to prepare electrocompetent cells, growth defects were observed with prolonged induction of lambda *red* and *recET* (Figure S1).

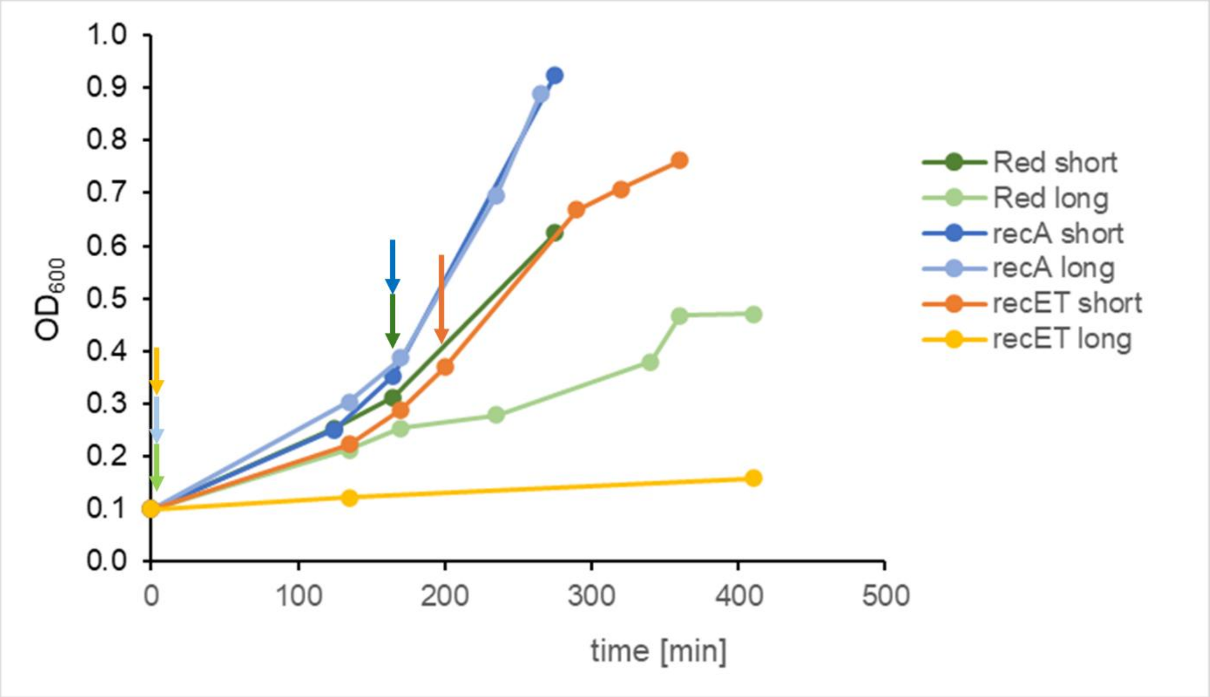

Figure S1. Growth behavior of *C. necator* during the preparation of electrocompetent cells under different induction protocols for lambda *red*, *recET*, and *C. necator recA*. The eGFP strains harboring the different recombineering systems were induced with 10 mM rhamnose at the indicated time points (arrows).

Electrocompetent cells prepared using each recombineering system were evaluated for transformation efficiency with unstable DNA vectors. Among the systems tested, sufficient transformation rates were observed only with the lambda Red recombineering system (Figure S2, A). Prolonged induction of lambda *red* reduced transformation rates by factors of approximately 6 and 13. Editing efficiencies, however, remained consistent at ~70–80% for plasmids and 90–100% for linear DNA fragments, regardless of whether lambda Red or *C. necator* RecA was employed (Figure S2, A and B).

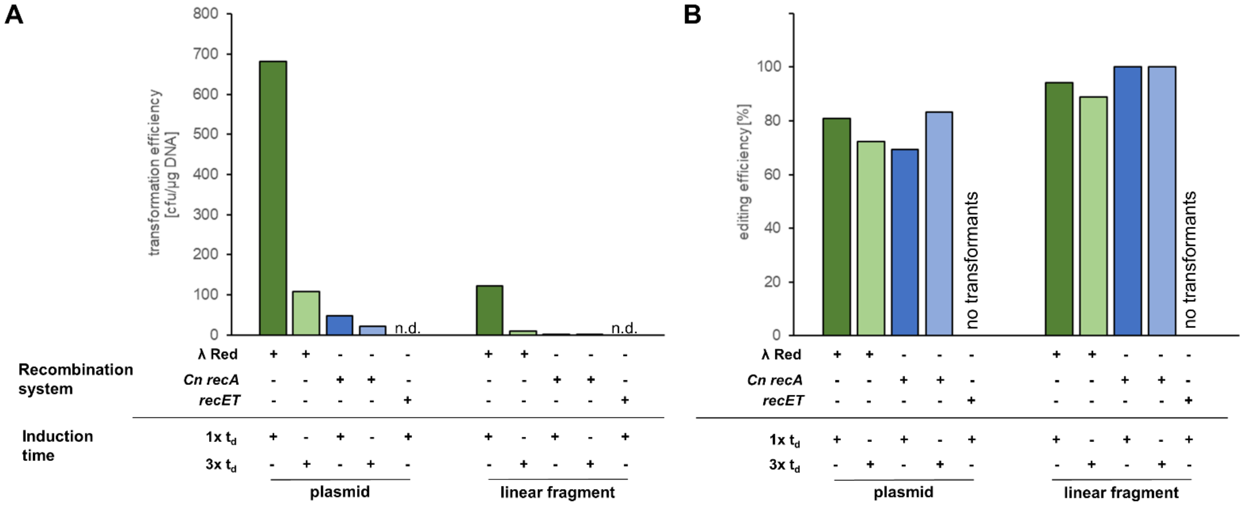

Figure S2: Transformation and editing efficiencies of the three tested recombineering systems in *C. necator*. Recombineering approaches were evaluated by targeting an integrated *eGFP* locus using an instable plasmid and a linear DNA fragment with 900 bp homologous arms. (A) Electroporation rates for each recombineering system. (B) Editing efficiencies determined by screening for the loss of fluorescence.

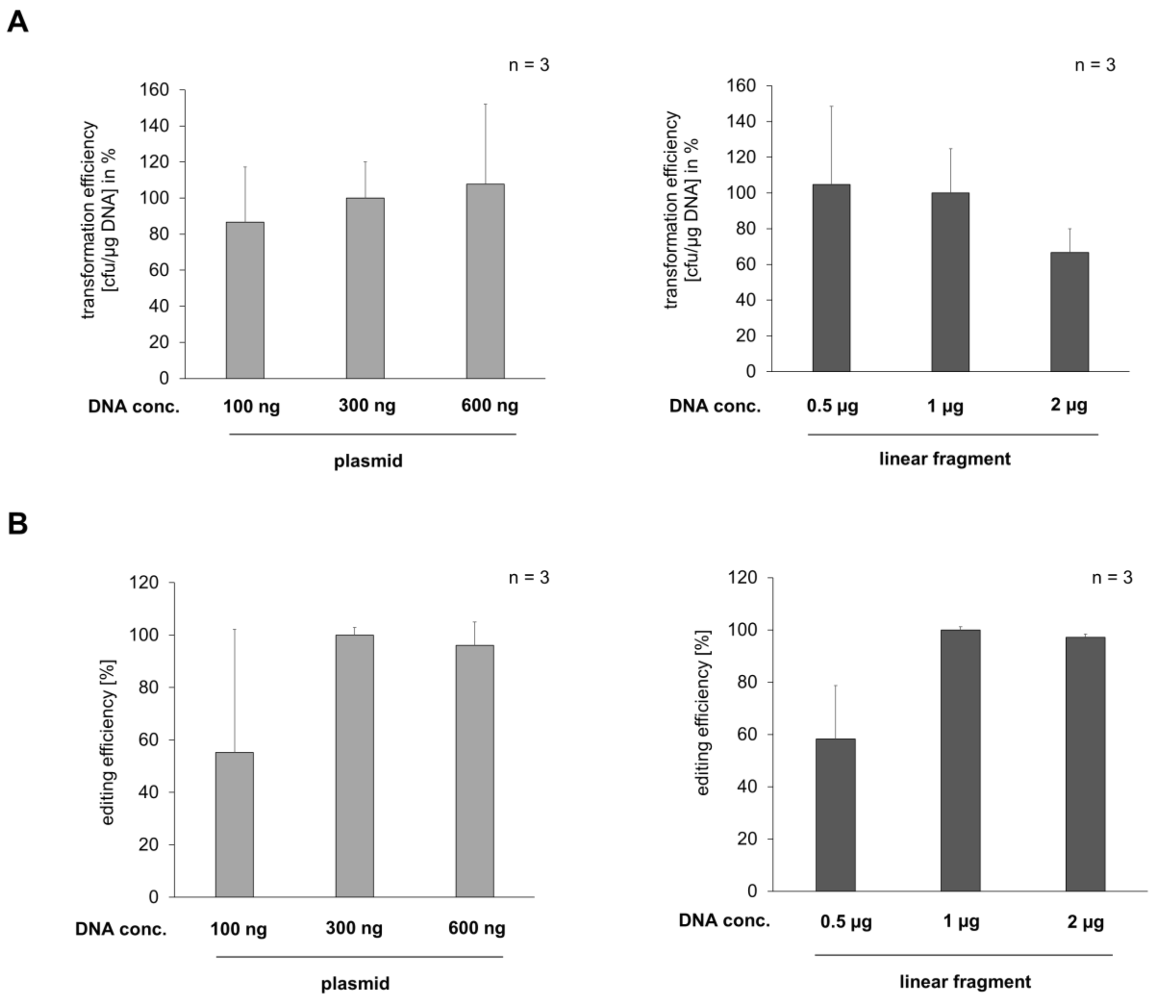

Figure S3. Comparison of transformation and editing efficiencies using varying DNA concentrations for lambda Red recombineering in *C. necator*. Recombineering efficiencies were evaluated by targeting an integrated *eGFP* locus with the suicide plasmid and linear DNA fragments featuring 900 bp homologous arms. (A) Transformation rates were assessed for different DNA concentrations, including 100, 300, and 600 ng of the suicide plasmid, as well as 0.5, 1, and 2 µg of the linear repair fragment. (B) Editing efficiencies of the transformants counted in (A) were determined by quantifying the loss of fluorescence, indicating successful editing events. Data from three transformations are presented.

The suicide plasmid pHInt-AppA-KanR-loxP was transformed into *C. necator* H16 wild-type, and transformants were selected on kanamycin-containing agar plates, as the goal was to integrate the cassette containing *appA* and Kan^R into the *phaC1* locus. Transformants were screened for correct integration by colony PCR using the primers listed in Table S2. The colony PCR results revealed the presence of the native *phaC1* locus in each clone, indicated by amplicons of 1.9 kb, along with other bands of undefined origin. In some clones, an additional band of 4.9 kb suggested a possible correct integration of the *appA* expression cassette (Figure S4). The consistent presence of the undisrupted wild-type locus in every clone indicates a common mechanism for avoiding selection for genomic integration via kanamycin, suggesting that the suicide plasmid might be maintained episomally.

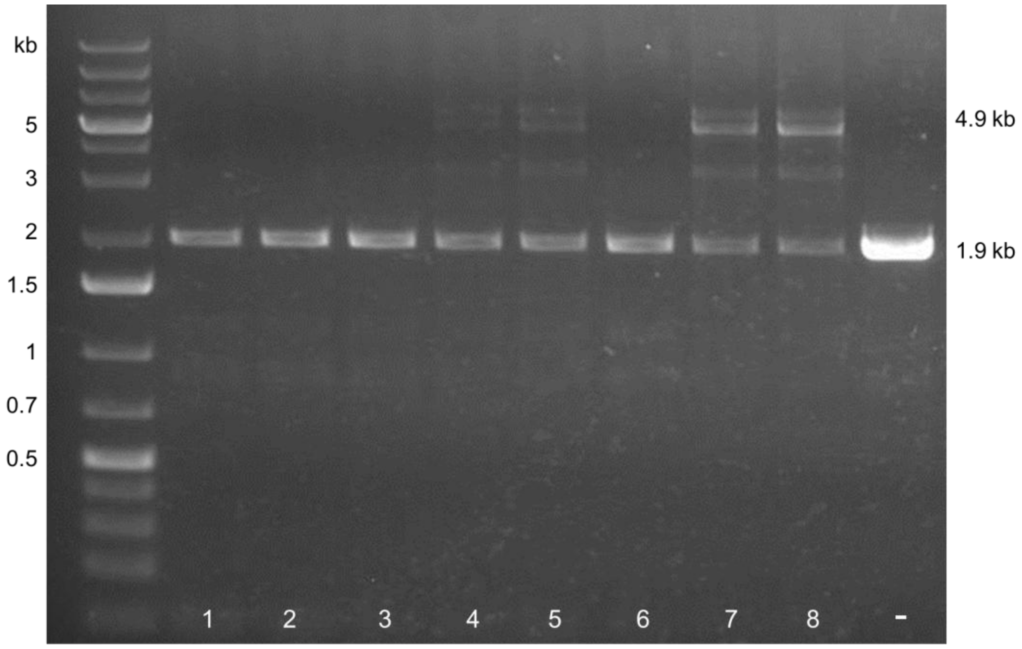

Figure S4: Colony PCR of *C. necator* H16 wild-type transformed with suicide plasmid pHInt-AppA-KanR-loxP. An integration of the *appA* expression cassette into the *phaC1* locus via double crossover leads to an amplicon of size of 4.9 kb. Bands of 1.9 kb in size correspond to the undisrupted wild type *phaC1*.

We therefore performed another colony PCR with primers designed to only give a product with circular plasmid DNA as template (primer pCont_BB_f1 and pSeq-ori-pLO3, see Table S10). Indeed, the corresponding agarose gel showed an amplicon of 4.9 kb for multiple clones, corresponding to the circular suicide plasmid (Figure S5).

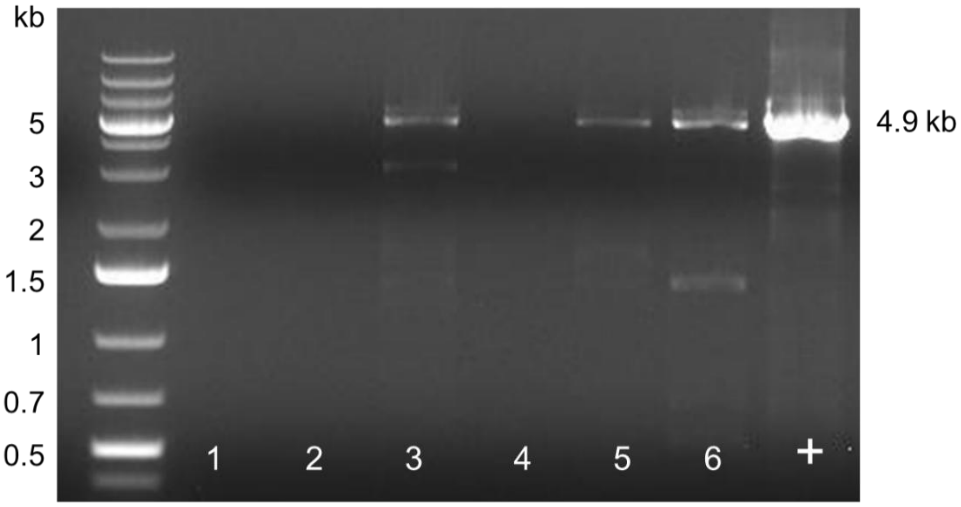

Figure S5: Circular plasmid-specific colony PCR of mutants transformed with pHInt-AppA-KanR-loxP. Primer amplifying a 4.9 kb fragment only in the presence of circular plasmid were chosen. (+) refers to the use of pHInt-AppA-KanR-loxP plasmid DNA as positive control.

As we aimed for a clear integration into the locus with *phaC1* disruption, we decided to proceed with the linear *appA* expression cassette to avoid strain instabilities. The linear fragment was transformed into *C. necator* H16 wildtype as well as the *eGFP* expression strain. Successful integration was verified with colony PCR using the primers listed in Table S11. Colony PCR results for multiple screened clones with a wild type (Figure S6) and an *eGFP* strain background (Figures S7) are shown below.

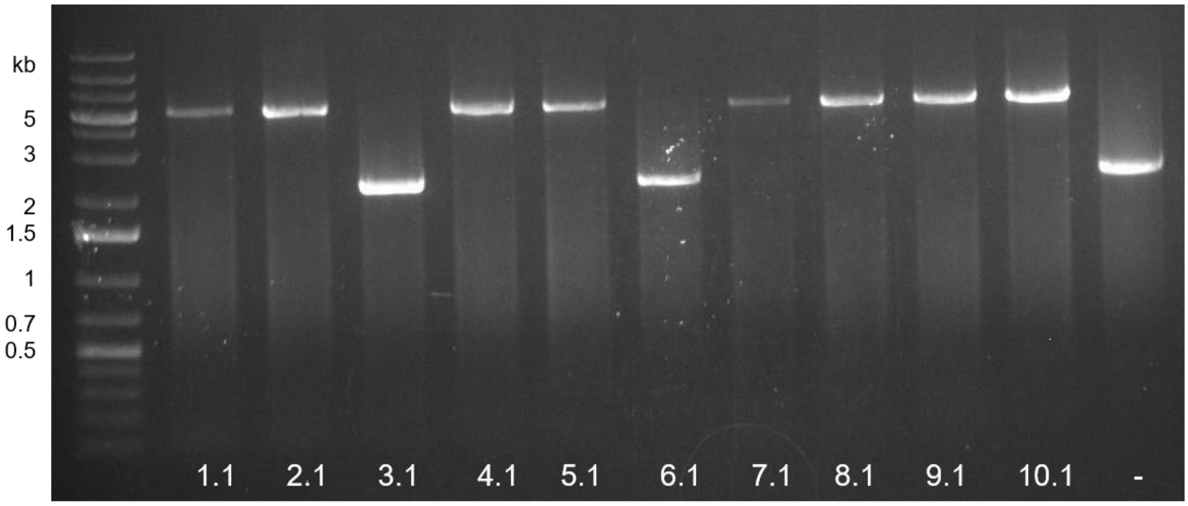

Figure S6: Control PCRs of *C. necator* WT transformed with the linear pHInt-AppA-PhaC-loxP fragment. Amplicons of 4.9 kb correspond to the target integration, amplicons at 1.9 kb correspond to the undisrupted native *phaC1* locus and therefore unsuccessful integration or negative control, respectively.

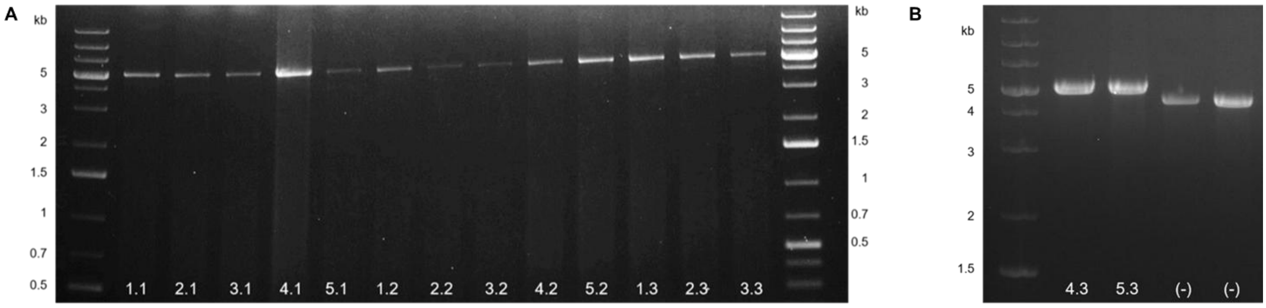

Figure S7: Control PCRs of *C. necator* eGFP transformed with the linear pHInt-AppA-PhaC-loxP fragment including negative control. The negative control correlates to the undisrupted eGFP locus with an amplicon size of approximately 4.3 kb while the integration of the *appA* cassette exhibits an amplicon of 4.9 kb.

To compare editing efficiency in relation to the length of target genes replaced or insert length, the observed results and the lengths of the corresponding DNA fragments are summarized in Figure S8.

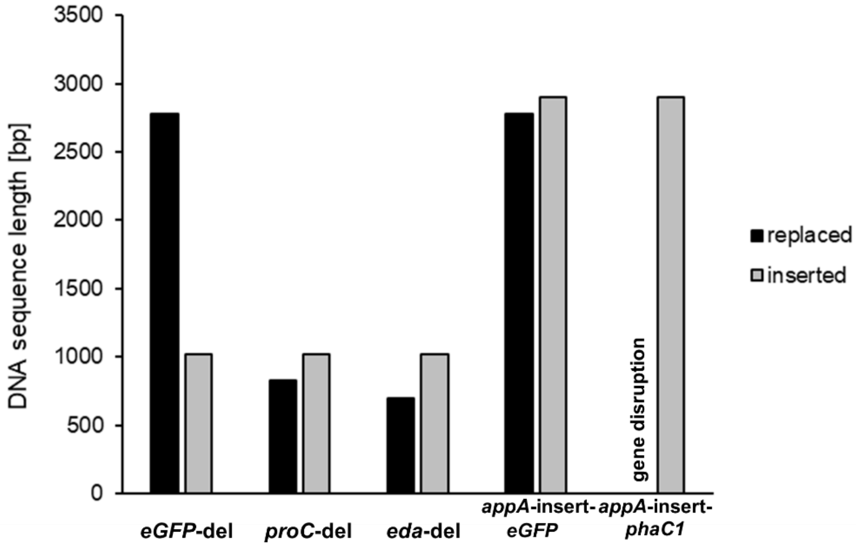

Editing efficiency		*eGFP*-del	*proC*-del	*eda*-del	*appA*-insert-*eGFP*	*appA*-insert-*phaC1*
	**plasmid**	60%	96%	89%	-	-
	**lin. DNA**	90%	92%	74%	31%	86%

Figure S8: Summary of editing efficiencies achieved by targeting three different loci and insertion of two different cassette sizes. The figure shows the length of the DNA region replaced by the applied strategies and the corresponding editing efficiencies for each insert size.

1.4 Plasmid sequences

>pCn-Red

ttctatgcgcacccgttctcggagcactgtccgaccgctttggccgccgcccagtcctgctcgcttcgctacttggagccactatcgactacgcgatcatggcgaccacacccgtcctgtggatcctctacgccgga
cgcatcgtggccggcatcaccggcgccacaggtgcggttgctggcgcctatatcgccgacatcaccgatggggaagatcgggctcgccacttcgggctcatgagcgcttgtttcggcgtgggtatggtggcag
gccccgtggccgggggactgttgggcgccatctccttgcatgcaccattccttgcggcggcggtgctcaacggcctcaacctactactgggctgcttcctaatgcaggagtcgcataagggagagcgtcgaccg
atgcccttgagagccttcaacccagtcagctccttccggtgggcgcggggcatgactatcgtcgccgcacttatgactgtcttctttatcatgcaactcgtaggacaggtgccggcagcgctctgggtcattttcggc
gaggaccgctttcgctggagcgcgacgatgatcggcctgtcgcttgcggtattcggaatcttgcacgccctcgctcaagccttcgtcactggtcccgccaccaaacgtttcggcgagaagcaggccattatcgcc
ggcatggcggccgacgcgctgggctacgtcttgctggcgttcgcgacgcgaggctggatggccttccccattatgattcttctcgcttccggcggcatcgggatgcccgcgttgcaggccatgctgtccaggcag
gtagatgacgaccatcagggacagcttcaaggatcgctcgcggctcttaccagcctaacttcgatcattggaccgctgatcgtcacggcgatttatgccgcctcggcgagcacatggaacgggttggcatggatt
gtaggcgccgccctataccttgtctgcctccccgcgttgcgtcgcggtgcatggagccgggccacctcgacctgaatggaagccggcggcacctcgctaacggattcaccactccaatttaaatatcacataaat
taattaaagatctcaaactgtacgtttaaacgctactttccgaacgactcctgcgttgatcggaaatccagaagcccgagaggttgccgcctttcgggctttttctttttcaaaaaaaaaaatttataaaacgatctgttg
cggccgccgggttgtgggcaaaggcgctcgacggtgggcaaccgcttgcggttgtccacgggcggagccggtgcgcgtagcgcattgtccacaagccaagggcgaccaataattgatatatatattcataatt
gaaaagctaattgaacatactacttgctgtaactacttgccggagcgaggggtgtttgcaagctgttgatctgaaagggctattagcgttctcacgtgccttttttgattagcgatttcacgtgaccttattagcgatttcac
gtactccgattagcgatttcacgtaccctgattagcgatttcacgtggatagtttttggagcgggccggaaagccccgtgaatcaaggctttgcggggcattagcggtttcacgtggataactaccctctatccacag
gcttccggggataaaaaagcccgctcgacggcgggctgttggatgggaaggcttgaccaagccaagcgtagcgttggcctggtcaagtcggagggggggccgatgcgagcgcccttgccgggtgcgcgggt
gacatgcaggcgtgtggatttgatgcgcaggcattcgccgtcatcttcgatgcagtcgcttgcctcgggatagacaatcaacacttcgcgtaggcgctttttgaagttgtatttgaagctggcgagtgctgcccgctct
gcccgctctcgggccttatcgtccagttcgggcgagttgcgtgcgcggctgccataggatgagccgaattgcgcttgcagggcgacccaagggatttgcacgaaggggcggcccttggcccgcaacaggaac
acgcgataggtcagccacgtgtaaatgtccatcgcaagcggagactgccgcaaggcatgcaggtagtcgattcggataggaaccggtgagcgggtgacttcctcgaagaaatcgcctgtgagggtgagggt
gctatcccatagcgcccgatcttctggccgcttgggattccagaatagaaaagcgcgcttggcaatgacgacgttctcaatgccgaagtcattgccttgctcgccggcaagcgaaatcatggatgaaaacaggc
gttgcgcctgattgcgaagggtggccgtgtaacggccatcggtgtgcattccgagcctttgtagaaattccgattgcgaccggccaaggttcaacacggggtctttcgttcgcacggcctcggtgcatatccaagc
aagcaaggtgcgcggcatagaaccgtagggcaggccgatgctcggcttgcccatgatcgacaaggtgacgatgccattggtgcgctcaaagtagctggtcttggggtcggtgtggggcatggtcgcttgcaca
aggcaacgggccatgtagccgactaagccagcttcgcgggcatcctccatttcgagcgcgaggctcgtcttgatgatctcgttgatacgatggccgggggctttgttgttcttaggcatgttgttccctccccggcat
ggtgatggttggtctagtgtttgtgggtttgatgttccggcgtttgatgaacaggcgcaaggtgtgagggctgacgcctaacaactcggctgcgcgactttgcggcaagccaaggttcacgtatgcctgtacttcatc
aatacggctgtccagcttcaaggcgctcgatttgctgcccttgggtcgcccgagcgtcttgccgcgctctctggcgacttgtagcgcctcggtggtacgtgcctgaatgaaatgccgctcgatctgtgcagccaagc
caagcacggttgccatgatgtcgctttgtaggctgccgtccatgatgatcttctgtttggtcacatggacgattaggccgcgctcgctcgccgctttgagaatttccaaggcggcgagggcggaaccggcaatgcg
cgtaatctccggcgtcagtagcacgtcgccacgctcggccttttcgatgattgctccgagcttgcgcttgcgccagtcctttgctctgctggcaatttcttcctcgatctgtagcggcgcgaagcctttggcgttcgcgtat
tcgagcaaaccgtatttttggttttccgggtcttggccgtcacgcgaaacccggagataggcatagtattttggcatttgcagggaaaacgtcagattcggttaaacatgcctcattctagcgcagattaaataggaa
ttaaataccctgttgcggtatagataaaacgttggtttgttctgccctatgagcgtacaaaaaaggccgggtgagtggcccggccttcgtttaggtgctgaataggattggttctggtctgccctgcaggtctagacgc
agaaaggcccacccgaaggtgagccagtgtgactctagtagtttaaacttaatctttctgcgaattgagatgacgccactggctgggcgtcatcccggtttcccgggtaaacaccaccgaaaaatagttactatct
tcaaagccacattcggtcgaaatatcactgattaacaggcggctatgctggagaagatattgcgcatgacacactctgacctgtcgcagatattgattgatggtcattccagtctgctggcgaaattgctgacgcaa
aacgcgctcactgcacgatgcctcatcacaaaatttatccagcgcaaagggacttttcaggctagccgccagccgggtaatcagcttatccagcaacgtttcgctggatgttggcggcaacgaatcactggtgta
acgatggcgattcagcaacatcaccaactgcccgaacagcaactcagccatttcgttagcaaacggcacatgctgactactttcatgctcaagctgaccgataacctgccgcgcctgcgccatccccatgctac
ctaagcgccagtgtggttgccctgcgctggcgttaaatcccggaatcgcccccctgccagtcaagattcagcttcagacgctccgggcaataaataatattctgcaaaaccagatcgttaacggaagcgtaggag
tgtttatcgtcagcatgaatgtaaaagagatcgccacgggtaatgcgataagggcgatcgttgagtacatgcaggccattaccgcgccagacaatcaccagctcacaaaaatcatgtgtatgttcagcaaagac
atcttgcggataacggtcagccacagcgactgcctgctggtcgctggcaaaaaaatcatctttgagaagttttaactgatgcgccaccgtggctacctcggccagagaacgaagttgattattcgcaatatggcgt
acaaatacgttgagaagattcgcgttattgcagaaagccatcccgtccctggcgaatatcacgcggtgaccagttaaactctcggcgaaaaagcgtcgaaaagtggttactgtcgctgaatccacagcgatag
gcgatgtcagtaacgctggcctcgctgtggcgtagcagatgtcgggctttcatcagtcgcaggcggttcaggtatcgctgaggcgtcagtcccgtttgctgcttaagctgccgatgtagcgtacgcagtgaaagag
aaaattgatccgccacggcatcccaattcacctcatcggcaaaatggtcctccagccaggccagaagcaagttgagacgtgatgcgctgttttccaggttctcctgcaaactgcttttacgcagcaagagcagta
attgcataaacaagatctcgcgactggcggtcgagggtaaatcattttccccttcctgctgttccatctgtgcaaccagctgtcgcacctgctgcaatacgctgtggttaacgcgccagtgagacggatactgcccat
ccagctcttgtggcagcaactgattcagcccggcgagaaactgaaatcgatccggcgagcgatacagcacattggtcagacacagattatcggtatgttcatacagatgccgatcatgatcgcgtacgaaaca
gaccgtgccaccggtgatggtatagggctgcccattaaacacatgaatacccgtgccatgttcgacaatcacaatttcatgaaaatcatgatgatgttcaggaaaatccgcctgcgggagccggggttctatcgc
cacggacgcgttaccagacggaaaaaaatccacactatgtaatacggtcatactggcctcctgatgtcgtcaacacggcgaaatagtaatcacgaggtcaggttcttaccttaaattttcgacggaaaaccacg
taaaaaacgtcgatttttcaagatacagcgtgaattttcaggaaatgcggtgagcatcacatcaccacaattcagcaaattgtgaacatcatcacgttcatctttccctggttgccaatggcccattttcctgtcagtaa
cgagaaggtcgcgaattcaggcgctttttagactggtcgtaatgaacaattcttaagaaggagatatacatatgagtactgcactcgcaacgctggctgggaagctggctgaacgtgtcggcatggattctgtcga
cccacaggaactgatcaccactcttcgccagacggcatttaaaggtgatgccagcgatgcgcagttcatcgcattactgatcgttgccaaccagtacggccttaatccgtggacgaaagaaatttacgcctttcct
gataagcagaatggcatcgttccggtggtgggcgttgatggctggtcccgcatcatcaatgaaaaccagcagtttgatggcatggactttgagcaggacaatgaatcctgtacatgccggatttaccgcaagga
ccgtaatcatccgatctgcgttaccgaatggatggatgaatgccgccgcgaaccattcaaaactcgcgaaggcagagaaatcacggggccgtggcagtcgcatcccaaacggatgttacgtcataaagccat
gattcagtgtgcccgtctggccttcggatttgctggtatctatgacaaggatgaagccgagcgcattgtcgaaaatactgcatacactgcagaacgtcagccggaacgcgacatcactccggttaacgatgaaa
ccatgcaggagattaacactctgctgatcgccctggataaaacatgggatgacgacttattgccgctctgttcccagatatttcgccgcgacattcgtgcatcgtcagaactgacacaggccgaagcagtaaaag
ctcttggattcctgaaacagaaagccgcagagcagaaggtggcagcatgaaaaggaggacaaccatgacaccggacattatcctgcagcgtaccgggatcgatgtgagagctgtcgaacagggggatga
tgcgtggcacaaattacggctcggcgtcatcaccgcttcagaagttcacaacgtgatagcaaaaccccgctccggaaagaagtggcctgacatgaaaatgtcctacttccacaccctgcttgctgaggtttgcac
cggtgtggctccggaagttaacgctaaagcactggcctggggaaaacagtacgagaacgacgccagaaccctgtttgaattcacttccggcgtgaatgttactgaatccccgatcatctatcgcgacgaaagt
atgcgtaccgcctgctctcccgatggtttatgcagtgacggcaacggccttgaactgaaatgcccgtttacctcccgggatttcatgaagttccggctcggtggtttcgaggccataaagtcagcttacatggccca
ggtgcagtacagcatgtgggtgacgcgaaaaaatgcctggtactttgccaactatgacccgcgtatgaagcgtgaaggcctgcattatgtcgtgattgagcgggatgaaaagtacatggcgagttttgacgaga
tcgtgccggagttcatcgaaaaaatggacgaggcactggctgaaattggttttgtatttggggagcaatggcgatgaaaaggaggacaaccatggatattaatactgaaactgagatcaagcaaaagcattca
ctaaccccctttcctgttttcctaatcagcccggcatttcgcgggcgatattttcacagctatttcaggagttcagccatgaacgcttattacattcaggatcgtcttgaggctcagagctgggcgcgtcactaccagca
gctcgcccgtgaagagaaagaggcagaactggcagacgacatggaaaaaggcctgccccagcacctgtttgaatcgctatgcatcgatcatttgcaacgccacggggccagcaaaaaaatccattacccgt
gcgtttgatgacgatgttgagtttcaggagcgcatggcagaacacatccggtacatggttgaaaccattgctcaccaccaggttgatattgattcagaggtataaagaaggccatcctgacggatggccttttccg
aaaagtgccacctgacgtctaagaaaccattattatcatgacattaacctataaaaataggcgtatcacgaggccctttcgtcttcaagaattctcatgtttgacagcttatcatcgataagctttaatgcggtagtttat
cacagttaaattgctaacgcagtcaggcaccgtgtatgaaatctaacaatgcgctcatcgtcatcctcggcaccgtcaccctggatgctgtaggcataggcttggttatgccggtactgccgggcctcttgcgggat
atcgtccattccgacagcatcgccagtcactatggcgtgctgctagcgctatatgcgttgatgcaat

>pCn-recA

ttctatgcgcacccgttctcggagcactgtccgaccgctttggccgccgcccagtcctgctcgcttcgctacttggagccactatcgactacgcgatcatggcgaccacacccgtcctgtggatcctctacgccgga
cgcatcgtggccggcatcaccggcgccacaggtgcggttgctggcgcctatatcgccgacatcaccgatggggaagatcgggctcgccacttcgggctcatgagcgcttgtttcggcgtgggtatggtggcag
gccccgtggccgggggactgttgggcgccatctccttgcatgcaccattccttgcggcggcggtgctcaacggcctcaacctactactgggctgcttcctaatgcaggagtcgcataagggagagcgtcgaccg
atgcccttgagagccttcaacccagtcagctccttccggtgggcgcggggcatgactatcgtcgccgcacttatgactgtcttctttatcatgcaactcgtaggacaggtgccggcagcgctctgggtcattttcggc
gaggaccgctttcgctggagcgcgacgatgatcggcctgtcgcttgcggtattcggaatcttgcacgccctcgctcaagccttcgtcactggtcccgccaccaaacgtttcggcgagaagcaggccattatcgcc
ggcatggcggccgacgcgctgggctacgtcttgctggcgttcgcgacgcgaggctggatggccttccccattatgattcttctcgcttccggcggcatcgggatgcccgcgttgcaggccatgctgtccaggcag
gtagatgacgaccatcagggacagcttcaaggatcgctcgcggctcttaccagcctaacttcgatcattggaccgctgatcgtcacggcgatttatgccgcctcggcgagcacatggaacgggttggcatggatt
gtaggcgccgccctataccttgtctgcctccccgcgttgcgtcgcggtgcatggagccgggccacctcgacctgaatggaagccggcggcacctcgctaacggattcaccactccaatttaaatatcacataaat
taattaaagatctcaaactgtacgtttaaacgctactttccgaacgactcctgcgttgatcggaaatccagaagcccgagaggttgccgcctttcgggctttttctttttcaaaaaaaaaaatttataaaacgatctgttg
cggccgccgggttgtgggcaaaggcgctcgacggtgggcaaccgcttgcggttgtccacgggcggagccggtgcgcgtagcgcattgtccacaagccaagggcgaccaataattgatatatatattcataatt
gaaaagctaattgaacatactacttgctgtaactacttgccggagcgaggggtgtttgcaagctgttgatctgaaagggctattagcgttctcacgtgccttttttgattagcgatttcacgtgaccttattagcgatttcac
gtactccgattagcgatttcacgtaccctgattagcgatttcacgtggatagtttttggagcgggccggaaagccccgtgaatcaaggctttgcggggcattagcggtttcacgtggataactaccctctatccacag
gcttccggggataaaaaagcccgctcgacggcgggctgttggatgggaaggcttgaccaagccaagcgtagcgttggcctggtcaagtcggagggggggccgatgcgagcgcccttgccgggtgcgcgggt
gacatgcaggcgtgtggatttgatgcgcaggcattcgccgtcatcttcgatgcagtcgcttgcctcgggatagacaatcaacacttcgcgtaggcgctttttgaagttgtatttgaagctggcgagtgctgcccgctct
gcccgctctcgggccttatcgtccagttcgggcgagttgcgtgcgcggctgccataggatgagccgaattgcgcttgcagggcgacccaagggatttgcacgaaggggcggcccttggcccgcaacaggaac
acgcgataggtcagccacgtgtaaatgtccatcgcaagcggagactgccgcaaggcatgcaggtagtcgattcggataggaaccggtgagcgggtgacttcctcgaagaaatcgcctgtgagggtgagggt
gctatcccatagcgcccgatcttctggccgcttgggattccagaatagaaaagcgcgcttggcaatgacgacgttctcaatgccgaagtcattgccttgctcgccggcaagcgaaatcatggatgaaaacaggc

gttgcgcctgattgcgaagggtggccgtgtaacggccatcggtgtgcattccgagcctttgtagaaattccgattgcgaccggccaaggttcaacacggggtctttcgttcgcacggcctcggtgcatatccaagc
aagcaaggtgcgcggcatagaaccgtagggcaggccgatgctcggcttgcccatgatcgacaaggtgacgatgccattggtgcgctcaaagtagctggtcttggggtcggtgtggggcatggtcgcttgcaca
aggcaacgggccatgtagccgactaagccagcttcgcgggcatcctccatttcgagcgcgaggctcgtcttgatgatctcgttgatacgatggccgggggctttgttgttcttaggcatgttgttccctccccggcat
ggtgatggttggtctagtgtttgtgggtttgatgttccggcgtttgatgaacaggcgcaaggtgtgagggctgacgcctaacaactcggctgcgcgactttgcggcaagccaaggttcacgtatgcctgtacttcatc
aatacggctgtccagcttcaaggcgctcgatttgctgcccttgggtcgcccgagcgtcttgccgcgctctctggcgacttgtagcgcctcggtggtacgtgcctgaatgaaatgccgctcgatctgtgcagccaagc
caagcacggttgccatgatgtcgctttgtaggctgccgtccatgatgatcttctgtttggtcacatggacgattaggccgcgctcgctcgccgctttgagaatttccaaggcggcgagggcggaaccggcaatgcg
cgtaatctccggcgtcagtagcacgtcgccacgctcggccttttcgatgattgctccgagcttgcgcttgcgccagtcctttgctctgctggcaatttcttcctcgatctgtagcggcgcgaagcctttggcgttcgcgtat
tcgagcaaaccgtatttttggttttccgggtcttggccgtcacgcgaaacccggagataggcatagtattttggcatttgcagggaaaacgtcagattcggttaaacatgcctcattctagcgcagattaaataggaa
ttaaataccctgttgcggtatagataaaacgttggtttgttctgccctatgagcgtacaaaaaaggccgggtgagtggcccggccttcgtttaggtgctgaataggattggttctggtctgccctgcaggtctagacgc
agaaaggcccacccgaaggtgagccagtgtgactctagtagtttaaacttaatctttctgcgaattgagatgacgccactggctgggcgtcatcccggtttcccgggtaaacaccaccgaaaaatagttactatct
tcaaagccacattcggtcgaaatatcactgattaacaggcggctatgctggagaagatattgcgcatgacacactctgacctgtcgcagatattgattgatggtcattccagtctgctggcgaaattgctgacgcaa
aacgcgctcactgcacgatgcctcatcacaaaatttatccagcgcaaagggacttttcaggctagccgccagccgggtaatcagcttatccagcaacgtttcgctggatgttggcggcaacgaatcactggtgta
acgatggcgattcagcaacatcaccaactgcccgaacagcaactcagccatttcgttagcaaacggcacatgctgactactttcatgctcaagctgaccgataacctgccgcgcctgcgccatccccatgctac
ctaagcgccagtgtggttgccctgcgctggcgttaaatcccggaatcgcccccctgccagtcaagattcagcttcagacgctccgggcaataaataatattctgcaaaaccagatcgttaacggaagcgtaggag
tgtttatcgtcagcatgaatgtaaaagagatcgccacgggtaatgcgataagggcgatcgttgagtacatgcaggccattaccgcgccagacaatcaccagctcacaaaaatcatgtgtatgttcagcaaagac
atcttgcggataacggtcagccacagcgactgcctgctggtcgctggcaaaaaaatcatctttgagaagttttaactgatgcgccaccgtggctacctcggccagagaacgaagttgattattcgcaatatggcgt
acaaatacgttgagaagattcgcgttattgcagaaagccatcccgtccctggcgaatatcacgcggtgaccagttaaactctcggcgaaaaagcgtcgaaaagtggttactgtcgctgaatccacagcgatag
gcgatgtcagtaacgctggcctcgctgtggcgtagcagatgtcgggctttcatcagtcgcaggcggttcaggtatcgctgaggcgtcagtcccgtttgctgcttaagctgccgatgtagcgtacgcagtgaaagag
aaaattgatccgccacggcatcccaattcacctcatcggcaaaatggtcctccagccaggccagaagcaagttgagacgtgatgcgctgttttccaggttctcctgcaaactgcttttacgcagcaagagcagta
attgcataaacaagatctcgcgactggcggtcgagggtaaatcattttccccttcctgctgttccatctgtgcaaccagctgtcgcacctgctgcaatacgctgtggttaacgcgccagtgagacggatactgcccat
ccagctcttgtggcagcaactgattcagcccggcgagaaactgaaatcgatccggcgagcgatacagcacattggtcagacacagattatcggtatgttcatacagatgccgatcatgatcgcgtacgaaaca
gaccgtgccaccggtgatggtatagggctgcccattaaacacatgaatacccgtgccatgttcgacaatcacaatttcatgaaaatcatgatgatgttcaggaaaatccgcctgcgggagccggggttctatcgc
cacggacgcgttaccagacggaaaaaaatccacactatgtaatacggtcatactggcctcctgatgtcgtcaacacggcgaaatagtaatcacgaggtcaggttcttaccttaaattttcgacggaaaaccacg
taaaaaacgtcgatttttcaagatacagcgtgaattttcaggaaatgcggtgagcatcacatcaccacaattcagcaaattgtgaacatcatcacgttcatctttccctggttgccaatggcccattttcctgtcagtaa
cgagaaggtcgcgaattcaggcgctttttagactggtcgtaatgaacaattcttaagaaggagatatacatatggacgacaagaaggcaggtgccggcgtgagcgccgagaagcagaaggcgcttgccgcc
gcgctctcccagatcgagaagcagttcggcaagggctcgatcatgcgcctgggcgacggcgaggtcgagaaggacattcaggtggtatccaccggttcgctcggcctggacatcgcgcttggcgtcggcgg
cctgccgcgcggccgcgtggtcgagatctatggtccggaatcgtcgggcaagaccacgctgacgctgcaggtcgtggccgagatgcagaagctgggcggcacctgcgcctttatcgacgccgagcatgcgc
tggacgtcagctacgccaacaagctgggcgtcaacgtcggcgacctgctgatctcgcagccggacaccggcgaacaggcgctggaaatcaccgacgcgctggtgcgctcgggctcgatcgacctgatcgt
gatcgactcggtggcggcgctggtgcccaaggccgaaatcgaaggcgaaatgggcgattcgctgcccggcctgcaggcccgcctgatgagccaggcgctgcgcaagctgaccggcaccatcaagcgca
ccaactgcctggtgatcttcatcaaccagatccgcatgaagatcggcgtgatgttcggctcgccggaaaccaccaccggcggcaacgcgctcaagttctacgcctcggtgcgcctggatatccgccgcatcgg
ctcgatcaagaagggcgacgacgtgatcggcaacgagaccaaggtcaaggtggtcaagaacaaggtctcgccgccgttccgcgaagccttcttcgacatcctctacggccagggcatctcgcgccagggc
gagatcatcgacctgggcgtggacgccaagatcgtcgagaagtccggcgcctggtacagctacaacggcgacaagatcggccagggcaaggacaacgcgcgcgaatacctgcgcgagaaccctgaca
tcgccgacgaaatcgagaacaaggtgcgcgcggcgctgggcgtggtggccatgaaccccacggcggctgccacgccggtagcggtcgaagactgaggcgcgccagaaggccatcctgacggatggcct
tttccgaaaagtgccacctgacgtctaagaaaccattattatcatgacattaacctataaaaataggcgtatcacgaggccctttcgtcttcaagaattctcatgtttgacagcttatcatcgataagctttaatgcggta
gtttatcacagttaaattgctaacgcagtcaggcaccgtgtatgaaatctaacaatgcgctcatcgtcatcctcggcaccgtcaccctggatgctgtaggcataggcttggttatgccggtactgccgggcctcttgc
gggatatcgtccattccgacagcatcgccagtcactatggcgtgctgctagcgctatatgcgttgatgcaat

>pCn-recET

ttctatgcgcacccgttctcggagcactgtccgaccgctttggccgccgcccagtcctgctcgcttcgctacttggagccactatcgactacgcgatcatggcgaccacacccgtcctgtggatcctctacgccgga
cgcatcgtggccggcatcaccggcgccacaggtgcggttgctggcgcctatatcgccgacatcaccgatggggaagatcgggctcgccacttcgggctcatgagcgcttgtttcggcgtgggtatggtggcag
gccccgtggccgggggactgttgggcgccatctccttgcatgcaccattccttgcggcggcggtgctcaacggcctcaacctactactgggctgcttcctaatgcaggagtcgcataagggagagcgtcgaccg
atgcccttgagagccttcaacccagtcagctccttccggtgggcgcggggcatgactatcgtcgccgcacttatgactgtcttctttatcatgcaactcgtaggacaggtgccggcagcgctctgggtcattttcggc
gaggaccgctttcgctggagcgcgacgatgatcggcctgtcgcttgcggtattcggaatcttgcacgccctcgctcaagccttcgtcactggtcccgccaccaaacgtttcggcgagaagcaggccattatcgcc
ggcatggcggccgacgcgctgggctacgtcttgctggcgttcgcgacgcgaggctggatggccttccccattatgattcttctcgcttccggcggcatcgggatgcccgcgttgcaggccatgctgtccaggcag
gtagatgacgaccatcagggacagcttcaaggatcgctcgcggctcttaccagcctaacttcgatcattggaccgctgatcgtcacggcgatttatgccgcctcggcgagcacatggaacgggttggcatggatt
gtaggcgccgccctataccttgtctgcctccccgcgttgcgtcgcggtgcatggagccgggccacctcgacctgaatggaagccggcggcacctcgctaacggattcaccactccaatttaaatatcacataaat
taattaaagatctcaaactgtacgtttaaacgctactttccgaacgactcctgcgttgatcggaaatccagaagcccgagaggttgccgcctttcgggctttttctttttcaaaaaaaaaaatttataaaacgatctgttg
cggccgccgggttgtgggcaaaggcgctcgacggtgggcaaccgcttgcggttgtccacgggcggagccggtgcgcgtagcgcattgtccacaagccaagggcgaccaataattgatatatatattcataatt
gaaaagctaattgaacatactacttgctgtaactacttgccggagcgaggggtgtttgcaagctgttgatctgaaagggctattagcgttctcacgtgccttttttgattagcgatttcacgtgaccttattagcgatttcac
gtactccgattagcgatttcacgtaccctgattagcgatttcacgtggatagtttttggagcgggccggaaagccccgtgaatcaaggctttgcggggcattagcggtttcacgtggataactaccctctatccacag
gcttccggggataaaaaagcccgctcgacggcgggctgttggatgggaaggcttgaccaagccaagcgtagcgttggcctggtcaagtcggagggggggccgatgcgagcgcccttgccgggtgcgcgggt
gacatgcaggcgtgtggatttgatgcgcaggcattcgccgtcatcttcgatgcagtcgcttgcctcgggatagacaatcaacacttcgcgtaggcgcttttttgaagttgtatttgaagctggcgagtgctgcccgctct
gcccgctctcgggccttatcgtccagttcgggcgagttgcgtgcgcggctgccataggatgagccgaattgcgcttgcagggcgacccaagggatttgcacgaaggggcggcccttggcccgcaacaggaac
acgcgataggtcagccacgtgtaaatgtccatcgcaagcggagactgccgcaaggcatgcaggtagtcgattcggataggaaccggtgagcgggtgacttcctcgaagaaatcgcctgtgagggtgagggt
gctatcccatagcgcccgatcttctggccgcttgggattccagaatagaaaagcgcgcttggcaatgacgacgttctcaatgccgaagtcattgccttgctcgccggcaagcgaaatcatggatgaaaacaggc
gttgcgcctgattgcgaagggtggccgtgtaacggccatcggtgtgcattccgagcctttgtagaaattccgattgcgaccggccaaggttcaacacggggtctttcgttcgcacggcctcggtgcatatccaagc
aagcaaggtgcgcggcatagaaccgtagggcaggccgatgctcggcttgcccatgatcgacaaggtgacgatgccattggtgcgctcaaagtagctggtcttggggtcggtgtggggcatggtcgcttgcaca
aggcaacgggccatgtagccgactaagccagcttcgcgggcatcctccatttcgagcgcgaggctcgtcttgatgatctcgttgatacgatggccgggggctttgttgttcttaggcatgttgttccctccccggcat
ggtgatggttggtctagtgtttgtgggtttgatgttccggcgtttgatgaacaggcgcaaggtgtgagggctgacgcctaacaactcggctgcgcgactttgcggcaagccaaggttcacgtatgcctgtacttcatc
aatacggctgtccagcttcaaggcgctcgatttgctgcccttgggtcgcccgagcgtcttgccgcgctctctggcgacttgtagcgcctcggtggtacgtgcctgaatgaaatgccgctcgatctgtgcagccaagc
caagcacggttgccatgatgtcgctttgtaggctgccgtccatgatgatcttctgtttggtcacatggacgattaggccgcgctcgctcgccgctttgagaatttccaaggcggcgagggcggaaccggcaatgcg
cgtaatctccggcgtcagtagcacgtcgccacgctcggccttttcgatgattgctccgagcttgcgcttgcgccagtcctttgctctgctggcaatttcttcctcgatctgtagcggcgcgaagcctttggcgttcgcgtat
tcgagcaaaccgtatttttggttttccgggtcttggccgtcacgcgaaacccggagataggcatagtattttggcatttgcagggaaaacgtcagattcggttaaacatgcctcattctagcgcagattaaataggaa
ttaaataccctgttgcggtatagataaaacgttggtttgttctgccctatgagcgtacaaaaaaggccgggtgagtggcccggccttcgtttaggtgctgaataggattggttctggtctgccctgcaggtctagacgc
agaaaggcccacccgaaggtgagccagtgtgactctagtagtttaaacttaatctttctgcgaattgagatgacgccactggctgggcgtcatcccggtttcccgggtaaacaccaccgaaaaatagttactatct
tcaaagccacattcggtcgaaatatcactgattaacaggcggctatgctggagaagatattgcgcatgacacactctgacctgtcgcagatattgattgatggtcattccagtctgctggcgaaattgctgacgcaa
aacgcgctcactgcacgatgcctcatcacaaaatttatccagcgcaaagggacttttcaggctagccgccagccgggtaatcagcttatccagcaacgtttcgctggatgttggcggcaacgaatcactggtgta
acgatggcgattcagcaacatcaccaactgcccgaacagcaactcagccatttcgttagcaaacggcacatgctgactactttcatgctcaagctgaccgataacctgccgcgcctgcgccatccccatgctac
ctaagcgccagtgtggttgccctgcgctggcgttaaatcccggaatcgcccccctgccagtcaagattcagcttcagacgctccgggcaataaataatattctgcaaaaccagatcgttaacggaagcgtaggag
tgtttatcgtcagcatgaatgtaaaagagatcgccacgggtaatgcgataagggcgatcgttgagtacatgcaggccattaccgcgccagacaatcaccagctcacaaaaatcatgtgtatgttcagcaaagac
atcttgcggataacggtcagccacagcgactgcctgctggtcgctggcaaaaaaatcatctttgagaagttttaactgatgcgccaccgtggctacctcggccagagaacgaagttgattattcgcaatatggcgt
acaaatacgttgagaagattcgcgttattgcagaaagccatcccgtccctggcgaatatcacgcggtgaccagttaaactctcggcgaaaaagcgtcgaaaagtggttactgtcgctgaatccacagcgatag
gcgatgtcagtaacgctggcctcgctgtggcgtagcagatgtcgggctttcatcagtcgcaggcggttcaggtatcgctgaggcgtcagtcccgtttgctgcttaagctgccgatgtagcgtacgcagtgaaagag
aaaattgatccgccacggcatcccaattcacctcatcggcaaaatggtcctccagccaggccagaagcaagttgagacgtgatgcgctgttttccaggttctcctgcaaactgcttttacgcagcaagagcagta
attgcataaacaagatctcgcgactggcggtcgagggtaaatcattttccccttcctgctgttccatctgtgcaaccagctgtcgcacctgctgcaatacgctgtggttaacgcgccagtgagacggatactgcccat
ccagctcttgtggcagcaactgattcagcccggcgagaaactgaaatcgatccggcgagcgatacagcacattggtcagacacagattatcggtatgttcatacagatgccgatcatgatcgcgtacgaaaca
gaccgtgccaccggtgatggtatagggctgcccattaaacacatgaatacccgtgccatgttcgacaatcacaatttcatgaaaatcatgatgatgttcaggaaaatccgcctgcgggagccggggttctatcgc
cacggacgcgttaccagacggaaaaaaatccacactatgtaatacggtcatactggcctcctgatgtcgtcaacacggcgaaatagtaatcacgaggtcaggttcttaccttaaattttcgacggaaaaccacg
taaaaaacgtcgatttttcaagatacagcgtgaattttcaggaaatgcggtgagcatcacatcaccacaattcagcaaattgtgaacatcatcacgttcatctttccctggttgccaatggcccattttcctgtcagtaa
cgagaaggtcgcgaattcaggcgctttttagactggtcgtaatgaacaattcttaagaaggagatatacatatgactaagcaaccaccaatcgcaaaagccgatctgcaaaaactcaggaaaccgtgcac
cagcagcagttaaaaatagcgacgtgattagttttattaaccagccatcaatgaaagagcaactggcagcagctcttccacgccatatgacggctgaacgtatgatccgtatcgccaccacagaaattcgtaaa
gttccggcgttaggaaactgtgacactatgagttttgtcagtgcgatcgtacagtgttcacagctcggacttgagccaggtagcgccctcggtcatgcatatttactgccttttggtaataaaaacgaaaagagcggt
aaaaagaacgttcagctaatcattggctatcgcggcatgattgatctggctcgccgttctggtcaaatcgccagcctgtcagcccgtgttgtccgtgaaggtgacgagtttagcttcgaatttggccttgatgaaaagt
taatacaccgcccgggagaaaacgaagatgccccggttacccacgtctatgctgtcgcaagactgaaagacggaggtactcagtttgaagttatgacgcgcaaacagattgagctggtgcgcagcctgagta
aagctggtaataacgggccgtgggtaactcactgggaagaaatggcaaagaaaacggctattcgtcgcctgttcaaatatttgcccgtatcaattgagatccagcgtgcagtatcaatggatgaaaaggaacc

actgacaatcgatcctgcagattcctctgtattaaccggggaatacagtgtaatcgataattcagaggaataaaaaggaggacaaccatgagcacaaaaccactcttcctgttacggaaagcgaaaaaatcat
ccggtgaacctgacgtcgtcctgtgggcaagcaacgattttgaatcgacctgtgccactctggactacctgatcgttaagtcaggtaaaaaactgagcagctattttaaagctgttgccacgaattttcctgtcgttaa
tgacctgcccgctgaaggtgagatcgattttacctggagtgaacgctatcaactcagcaaagactccatgacatgggaactaaaaccgggagcagcaccagacaacgctcactatcaaggcaataccaacg
tcaacggcgaagacatgactgagattgaggagaatatgctactcccaatttctggccaggaactgcccattcgttggcttgctcaacacggcagcgaaaaaccggtaacgcacgtttcacgcgacggactcca
ggcattacacattgctcgggctgaagaactaccggctgttactgccctggctgtttcccacaaaaccagcctgctcgacccgctggaaattcgcgaactccacaaactggttcgtgacactgacaaagttttccct
aatcctggtaattcaaacctgggactgataactgctttttcgaagcatacctgaacgctgactacaccgatcgaggactgctgacaaaagagtggatgaagggtaatcgtgtttcacacatcactcgcacggctt
ccggtgctaatgctggcggcggaaacctcaccgatcgcggcgaaggtttcgtacacgatctgacgtcactggcgcgcgacgtagccactggcgtactggcccgttcaatggatctggacatctataaccttcatc
cggcacacgctaaacgcattgaggaaattatcgctgaaaataaaccgcccttttctgttttccgcgacaaattcatcaccatgcctggcgggctggattattcccgcgccatcgtggttgcgtccgtaaaagaagc
accaattgggatcgaggtcatccccgcgcacgtcactgaatatctgaacaaagtactgactgaaaccgatcatgccaaccctgatccggaaatcgtggatattgcctgcggtcgctcctctgccccgatgccgc
agcgagtaacagaagaaggaaaacaggatgatgaagaaaaaccgcaaccatctggaacaacggcagttgaacagggagaggctgaaacaatggaaccggacgcaactgaacatcatcaggacacg
cagccgctggatgctcagtcacaggtaaattctgttgatgcgaaatatcaggaactgcgggcagaactccatgaagcccggaaaaacattccatcaaaaaatcctgtcgatgacgataaattgcttgctgcatc
acgtggtgaatttgttgacggaattagcgacccgaacgatccgaaatgggtaaaggggatccagactcgcgattgtgtgtaccagaaccagccagaaacggaaaaaaccagcccagatatgaatcaacct
gagccagtagtgcaacaggaaccggaaatagcctgcaatgcctgcggccagactggcggggataactgccctgactgtggtgcggtgatgggcgacgcaacataccaggaaacattcgatgaagagagt
caggttgaagctaaggaaaatgatccggaggaaatggaaggcgctgaacatccgcacaatgagaatgctggcagcgatccgcatcgcgattgcagtgatgaaactggcgaagtcgcagatcccgtaatcg
tagaagacatagagccaggtatttattacggaatttcgaatgagaattaccacgcgggtcccggtatcagtaagtctcagctcgatgacattgctgatactccggcactatatttgtggcgtaaaaatgcccccgtg
gacaccacaaagacaaaaacgctcgatttaggaactgctttccactgccgggtacttgaaccggaagaattcagtaaccgctttatcgtagcacctgaatttaaccgccgtacaaacgccggaaaagaagaa
gagaaagcgtttctgatggaatgcgcaagcacaggaaaaacggttatcactgcggaagaaggccggaaaattgaactcatgtatcaaagcgttatggctttgccgctggggcaatggcttgttgaaagcgccg
gacacgctgaatcatcaatttactgggaagatcctgaaacaggaattttgtgtcggtgccgtccggacaaaattatccctgaatttcactggatcatggacgtgaaaactacggcggatattcaacgattcaaaac
cgcttattacgactaccgctatcacgttcaggatgcattctacagtgacggttatgaagcacagtttggagtgcagccaactttcgtttttctggttgccagcacaactattgaatgcggacgttatccggttgaaattttc
atgatgggcgaagaagcaaaactggcaggtcaacaggaatatcaccgcaatctgcgaaccctgtctgactgcctgaataccgatgaatggccagctattaagacattatcactgccccgctgggctaaggaa
tatgcaaatgactaaagaaggccatcctgacggatggccttttccgaaaagtgccacctgacgtctaagaaaccattattatcatgacattaacctataaaaataggcgtatcacgaggccctttcgtcttcaaga
attctcatgtttgacagcttatcatcgataagctttaatgcggtagtttatcacagttaaattgctaacgcagtcaggcaccgtgtatgaaatctaacaatgcgctcatcgtcatcctcggcaccgtcaccctggatgctg
taggcataggcttggttatgccggtactgccgggcctcttgcgggatatcgtccattccgacagcatcgccagtcactatggcgtgctgctagcgctatatgcgttgatgcaat

>pHRep-HKH1

tggtcccgccagtggcagggcactgaaggcaacggccacgcggccgcgtccggcattccgggcctggatgcgctggcaggcgtcaagatcgcgccggcgcagctgggtgatatccagcagcgctacatg
aaggacttctcagcgctgtggcaggccatggccgagggcaaggccgaggccaccggtccgctgcacgaccggcgcttcgccggcgacgcatggcgcaccaacctcccatatcgcttcgctgccgcgttcta
cctgctcaatgcgcgcgccttgaccgagctggccgatgccgtcgaggccgatgccaagacccgccagcgcatccgcttcgcgatctcgcaatgggtcgatgcgatgtcgcccgccaacttccttgccaccaat
cccgaggcgcagcgcctgctgatcgagtcgggcggcgaatcgctgcgtgccggcgtgcgcaacatgatggaagacctgacacgcggcaagatctcgcagaccgacgagagcgcgtttgaggtcggccgc
aatgtcgcggtgaccgaaggcgccgtggtcttcgagaacgagtacttccagctgttgcagtacaagccgctgaccgacaaggtgcacgcgcgcccgctgctgatggtgccgccgtgcatcaacaagtactac
atcctggacctgcagccggagagctcgctggtgcgccatgtggtggagcagggacatacggtgtttctggtgtcgtggcgcaatccggacgccagcatggccggcagcacctgggacgactgaattctttgcgt
ttctacaaactcttttgtttatttttctaaatacattcaatatgtatccgctcatgaacaataaaactgtctgcttacataaacagtaatacaaggggtgttatgagccatattcaacgggaaacgtcttgctctaggccgc
gattaaattccaacatggatgctgatttatatgggtataaatgggctcgcgataatgtcgggcaatcaggtgcgacaatctatcgattgtatgggaagcccgatgcgccagagttgtttctgaaacatggcaaaggt
agcgttgccaatgatgttacagatgagatggtcagactaaactggctgacggaatttatgcctcttccgaccatcaagcattttatccgtactcctgatgatgcatggttactcaccactgcgatccccgggaaaaca
gcattccaggtattagaagaatatcctgattcaggtgaaaatattgttgatgcgctggcagtgttcctgcgccggttgcattcgattcctgtttgtaattgtccttttaacagcgatcgcgtatttcgtctcgctcaggcgca
atcacgaatgaataacggtttggttgatgcgagtgattttgatgacgagcgtaatggctggcctgttgaacaagtctggaaagaaatgcataaacttttgccattctcaccggattcagtcgtcactcatggtgatttct
cacttgataaccttatttttgacgaggggaaattaataggttgtattgatgttggacgagtcggaatcgcagaccgataccaggatcttgccatcctatggaactgcctcggtgagttttctccttcattacagaaacgg
ctttttcaaaaatatggtattgataatcctgatatgaataaattgcagtttcatttgatgctcgatgagtttttctaagaattaattcatgaccaaaatcccttaacgtgagttttcgttccactgagcgtcagacaggcctcct
aggacggatggccttggatccacatcgagcacgcggccatccgcgccatcgaagtcgcgcgcgacatcagcggccaggacaagatcaacgtgctcggcttctgcgtgggcggcaccattgtctcgaccgc
gctggcggtgctggccgcgcgcggcgagcacccggccgccagcgtcacgctgctgaccacgctgctggactttgccgacacgggcatcctcgacgtctttgtcgacgagggccatgtgcagttgcgcgaggc
cacgctgggcggcggcgccggcgcgccgtgcgcgctgctgcgcggccttgagctggccaataccttctcgttcttgcgcccgaacgacctggtgtggaactacgtggtcgacaactacctgaagggcaacac
gccggtgccgttcgacctgctgttctggaacggcgacgccaccaacctgccggggccgtggtactgctggtacctgcgccacacctacctgcagaacgagctcaaggtaccgggcaagctgaccgtgtgcgg
cgtgccggtggacctggccagcatcgacgtgccgacctatatctacggctcgcgcgaagaccatatcgtgccgtggaccgcggcctatgcctcgaccgcgctgctggcgaacaagctgcgcttcgtgctgggt
gcgtcgggccatatcgccggtgtgatcaacccgccggccaagaacaagcgcagccactggactaacgatgcgctgccggagtcgccgcagcaatggctggccggcgccatcgagcatcacggcagctg
gtggccggactggaccgcatggctggccgggcaggccggcgcgaaacgcgccgcgcccgccaactatggcaatgcgcgctatcgcgcaatcgaacccgcgcctgggcgatacgtcaaagccaaggcat
gactcgagatgtgagcaaaaggccagcaaaaggccaggaaccgtaaaaaggccgcgttgctggcgtttttccataggctccgcccccctgacgagcatcacaaaaatcgacgctcaagtcagaggtggcg
aaacccgacaggactataaagataccaggcgtttccccctggaagctccctcgtgcgctctcctgttccgaccctgccgcttaccggatacctgtccgcctttctcccttcgggaagcgtggcgctttctcatagctc
acgctgtaggtatctcagttcggtgtaggtcgttcgctccaagctgggctgtgtgcacgaaccccccgttcagcccgaccgctgcgccttatccggtaactatcgtcttgagtccaacccggtaagacacgacttat
cgccactggcagcagccactggtaacaggattagcagagcgaggtatgtaggcggtgctacagagttcttgaagtggtggcctaactacggctacactagaaggacagtatttggtatctgcgctctgctgaag
ccagttaccttcggaaaaagagttggtagctcttgatccggcaaacaaaccaccgctggtagcggtggtttttttgtttgcaagcagcagattacgcgcagaaaaaaaggatctcaagaagatcctttgatcttttct
acgggtctagaatggcgaccggcaaaggcgcggcagcttccacgcaggaaggcaagtcccaaccattcaaggtcacgccggggccattcgatccagccacatggctggaa

>pHInt-AppA-KanR-loxP

tggtcccgccagtggcagggcactgaaggcaacggccacgcggccgcgtccggcattccgggcctggatgcgctggcaggcgtcaagatcgcgccggcgcagctgggtgatatccagcagcgctacatg
aaggacttctcagcgctgtggcaggccatggccgagggcaaggccgaggccaccggtccgctgcacgaccggcgcttcgccggcgacgcatggcgcaccaacctcccatatcgcttcgctgccgcgttcta
cctgctcaatgcgcgcgccttgaccgagctggccgatgccgtcgaggccgatgccaagacccgccagcgcatccgcttcgcgatctcgcaatgggtcgatgcgatgtcgcccgccaacttccttgccaccaat
cccgaggcgcagcgcctgctgatcgagtcgggcggcgaatcgctgcgtgccggcgtgcgcaacatgatggaagacctgacacgcggcaagatctcgcagaccgacgagagcgcgtttgaggtcggccgc
aatgtcgcggtgaccgaaggcgccgtggtcttcgagaacgagtacttccagctgttgcagtacaagccgctgaccgacaaggtgcacgcgcgcccgctgctgatggtgccgccgtgcatcaacaagtactac
atcctggacctgcagccggagagctcgctggtgcgccatgtggtggagcagggacatacggtgtttctggtgtcgtggcgcaatccggacgccagcatggccggcagcacctgggacgactgcatgcaaaa
accgttattgacacaggtggaaatttagaatatactgaagctttttgtttaactttaagaaggagatataggatccatgaaagcgatcttaatcccatttttatctcttctgattccgttaaccccgcaatctgcattcgctc
agagtgagccggagctgaagctggaaagtgtggtgattgtcagtcgtcatggtgtgcgtgctccaaccaaggccacgcaactgatgcaggatgtcaccccagacgcatggccaacctggccggtaaaactg
ggttggctgacaccgcgcggtggtgagctaatcgcctatctcggacattaccaacgccagcgtctggtagccgacggattgctggcgaaaaagggctgcccgcagtctggtcaggtcgcgattattgctgatgtc
gacgagcgtacccgtaaaacaggcgaagccttcgccgccgggctggcacctgactgtgcaataaccgtacatacccaggcagatacgtccagtcccgatccgttatttaatcctctaaaaactggcgtttgcca
actggataacgcgaacgtgactgacgcgatcctcagcagggcaggagggtcaattgctgactttaccgggcatcggcaaacggcgtttcgcgaactggaacgggtgcttaattttccgcaatcaaacttgtgcc
ttaaacgtgagaaacaggacgaaagctgttcattaacgcaggcattaccatcggaactcaaggtgagcgccgacaatgtctcattaaccggtgcggtaagcctcgcatcaatgctgacggagatatttctcctg
caacaagcacagggaatgccggagccggggtggggaaggatcaccgattcacaccagtggaacaccttgctaagtttgcataacgcgcaattttatttgctacaacgcacgccagaggttgcccgcagccg
cgccaccccgttattagatttgatcaagacagcgttgacgccccatccaccgcaaaaacaggcgtatggtgtgacattacccacttcagtgctgtttatcgccggacacgatactaatctggcaaatctcggcggc
gcactggagctcaactggacgcttcccggtcagccggataacacgccgccaggtggtgaactggtgtttgaacgctggcgtcggctaagcgataacagccagtggattcaggtttcgctggtcttccagacttta
cagcagatgcgtgataaaacgccgctgtcattaaatacgccgcccggagaggtgaaactgaccctggcaggatgtgaagagcgaaatgcgcagggcatgtgttcgttggcaggttttacgcaaatcgtgaat
gaagcacgcataccggcgtgcagtttgcatcaccatcaccatcactaagaattcggctgttttggcggatgagagaagattttcagcctgatacagattaaatcagaacgcagaagcggtctgataaaacaga
atttgcctggcggcagtagcgcggtggtcccacctgaccccatgccgaactcagaagtgaaacgccgtagcgccgatggtagtgtggggtctccccatgcgagagtagggaactgccaggcatcaaataaa
acgaaaggctcagtcgaaagactgggcctttcgttttatctgttgtttgtcggtgaacgctctcctgagtaggacaaatccgccgggagcggatttgaacgttgcgaagcaacggcccggagggtggcgggcag
gacgcccgccataaactgccaggcatcaaattaagcagaaggccatcctgacggatggcctttttaattaaataacttcgtataatgtatgctatacgaagttatcatatgtttgcgtttctacaaactcttttgtttatttttc
taaatacattcaatatgtatccgctcatgaacaataaaactgtctgcttacataaacagtaatacaaggggtgttatgagccatattcaacgggaaacgtcttgctctaggccgcgattaaattccaacatggatgct
gatttatatgggtataaatgggctcgcgataatgtcgggcaatcaggtgcgacaatctatcgattgtatgggaagcccgatgcgccagagttgtttctgaaacatggcaaaggtagcgttgccaatgatgttacag
atgagatggtcagactaaactggctgacggaatttatgcctcttccgaccatcaagcattttatccgtactcctgatgatgcatggttactcaccactgcgatccccgggaaaacagcattccaggtattagaagaa
tatcctgattcaggtgaaaatattgttgatgcgctggcagtgttcctgcgccggttgcattcgattcctgtttgtaattgtccttttaacagcgatcgcgtatttcgtctcgctcaggcgcaatcacgaatgaataacggtttg
gttgatgcgagtgattttgatgacgagcgtaatggctggcctgttgaacaagtctggaaagaaatgcataaacttttgccattctcaccggattcagtcgtcactcatggtgatttctcacttgataaccttatttttgacg
aggggaaattaataggttgtattgatgttggacgagtcggaatcgcagaccgataccaggatcttgccatcctatggaactgcctcggtgagttttctccttcattacagaaacggctttttcaaaaatatggtattgat
aatcctgatatgaataaattgcagtttcatttgatgctcgatgagtttttctaagaattaattcatgaccaaaatcccttaacgtgagttttcgttccactgagcgtcagacaggcctcctaggacggatggccttactagt
ataacttcgtataatgtatgctatacgaagttatacatcgagcacgcggccatccgcgccatcgaagtcgcgcgcgacatcagcggccaggacaagatcaacgtgctcggcttctgcgtgggcggcaccattgt

ctcgaccgcgctggcggtgctggccgcgcgcggcgagcacccggccgccagcgtcacgctgctgaccacgctgctggactttgccgacacgggcatcctcgacgtctttgtcgacgagggccatgtgcagtt
gcgcgaggccacgctgggcggcggcgccggcgcgccgtgcgcgctgctgcgcggccttgagctggccaataccttctcgttcttgcgcccgaacgacctggtgtggaactacgtggtcgacaactacctgaa
gggcaacacgccggtgccgttcgacctgctgttctggaacggcgacgccaccaacctgccggggccgtggtactgctggtacctgcgccacacctacctgcagaacgagctcaaggtaccgggcaagctga
ccgtgtgcggcgtgccggtggacctggccagcatcgacgtgccgacctatatctacggctcgcgcgaagaccatatcgtgccgtggaccgcggcctatgcctcgaccgcgctgctggcgaacaagctgcgctt
cgtgctgggtgcgtcgggccatatcgccggtgtgatcaacccgccggccaagaacaagcgcagccactggactaacgatgcgctgccggagtcgccgcagcaatggctggccggcgccatcgagcatca
cggcagctggtggccggactggaccgcatggctggccgggcaggccggcgcgaaacgcgccgcgcccgccaactatggcaatgcgcgctatcgcgcaatcgaacccgcgcctgggcgatacgtcaaag
ccaaggcatgactcgagatgtgagcaaaaggccagcaaaaggccaggaaccgtaaaaaggccgcgttgctggcgtttttccataggctccgcccccctgacgagcatcacaaaaatcgacgctcaagtca
gaggtggcgaaacccgacaggactataaagataccaggcgtttccccctggaagctccctcgtgcgctctcctgttccgaccctgccgcttaccggatacctgtccgcctttctcccttcgggaagcgtggcgcttt
ctcatagctcacgctgtaggtatctcagttcggtgtaggtcgttcgctccaagctgggctgtgtgcacgaaccccccgttcagcccgaccgctgcgccttatccggtaactatcgtcttgagtccaacccggtaagac
acgacttatcgccactggcagcagccactggtaacaggattagcagagcgaggtatgtaggcggtgctacagagttcttgaagtggtggcctaactacggctacactagaaggacagtatttggtatctgcgctc
tgctgaagccagttaccttcggaaaaagagttggtagctcttgatccggcaaacaaaccaccgctggtagcggtggtttttttgtttgcaagcagcagattacgcgcagaaaaaaaggatctcaagaagatccttt
gatcttttctacgggtctagaatggcgaccggcaaaggcgcggcagcttccacgcaggaaggcaagtcccaaccattcaaggtcacgccggggccattcgatccagccacatggctggaa

>pCn-Cre

atgtgagcaaaaggccagcaaaaggccaggaaccgtaaaaaggccgcgttgctggcgtttttccataggctccgcccccctgacgagcatcacaaaaatcgacgctcaagtcagaggtggcgaaacccg
acaggactataaagataccaggcgtttccccctggaagctccctcgtgcgctctcctgttccgaccctgccgcttaccggatacctgtccgcctttctcccttcgggaagcgtggcgctttctcatagctcacgctgta
ggtatctcagttcggtgtaggtcgttcgctccaagctgggctgtgtgcacgaaccccccgttcagcccgaccgctgcgccttatccggtaactatcgtcttgagtccaacccggtaagacacgacttatcgccactg
gcagcagccactggtaacaggattagcagagcgaggtatgtaggcggtgctacagagttcttgaagtggtggcctaactacggctacactagaaggacagtatttggtatctgcgctctgctgaagccagttac
cttcggaaaaagagttggtagctcttgatccggcaaacaaaccaccgctggtagcggtggtttttttgtttgcaagcagcagattacgcgcagaaaaaaaggatctcaagaagatcctttgatcttttctacggggt
cgacacggatggcctttttgcgtttctacaaactcttttgtttatttttctaaatacattcaaatatgtatccgctcatgaacaataaaactgtctgcttacataaacattgaaaaaggaagagtatgagtccaaagaga
agaacacaggcagagcgcgcaatggagacccagggcaagttgattgcagcggccctggggggttttacgggaaaaaggttacgcgggattccggatcgcagatgtgcccggtgctgcaggtgtctcgagag
gagcgcagagccatcatttcccgacaaagcttgagcttctgcttgccacttttgaatggctttacgaacagatcaccgaacgcagtcgggctcgattagcgaaattgaagccagaggatgacgtcatccagcaa
atgctggacgacgccgccgaatttttcctcgacgatgacttctctatcagccttgatttgattgtggctgccgaccgggatccagcgttacgcgagggtattcagcgcacggtagagaggaatcggtttgtcgtcga
ggatatgtggcttggtgttctggtgagccgtggtctttcgcgtgatgatgcagaagatatcctttggttgatattcaattcggtgcgtgggcttgctgttcgtagcctatggcagaaggacaaagaacgctttgagcgtgt
caggaactcgacactcgaaattgcgcgagagcggtacgcgaaattcaagcgctagcaaaaaacccctcaagacccgtttagaggccccaaggggttatgctagaatgagctgttgacaattaatcatcggct
cgtataatgtgtggaacaaacagacaatctggtctgtttgtattatagaattcgagctcggtacccggggatcctctagaaataattttgtttaactttaagaaggagatatacatatgtccaatttactgaccgtacacc
aaaatttgcctgcattaccggtcgatgcaacgagtgatgaggttcgcaagaacctgatggacatgttcagggatcgccaggcgttttctgagcatacctggaaaatgcttctgtccgtttgccggtcgtgggcggca
tggtgcaagttgaataaccggaaatggtttcccgcagaacctgaagatgttcgcgattatcttctatatcttcaggcgcgcggtctggcagtaaaaactatccagcaacatttgggccagctaaacatgcttcatcgt
cggtccgggctgccacgaccaagtgacagcaatgctgtttcactggttatgcggcggatccgaaaagaaaacgttgatgccggtgaacgtgcaaaacaggctctagcgttcgaacgcactgatttcgaccag
gttcgttcactcatggaaaatagcgatcgctgccaggatatacgtaatctggcatttctggggattgcttataacaccctgttacgtatagccgaaattgccaggatcagggttaaagatatctcacgtactgacggt
gggagaatgttaatccatattggcagaacgaaaacgctggttagcaccgcaggtgtagagaaggcacttagcctgggggtaactaaactggtcgagcgatggatttccgtctctggtgtagctgatgatccgaat
aactacctgttttgccgggtcagaaaaaatggtgttgccgcgccatctgccaccagccagctatcaactcgcgccctggaagggatttttgaagcaactcatcgattgatttacggcgctaaggatgactctggtca
gagatacctggcctggtctggacacagtgcccgtgtcggagccgcgcgagatatggcccgcgctggagtttcaataccggagatcatgcaagctggtggctggaccaatgtaaatattgtcatgaactatatcc
gtaacctggatagtgaaacaggggcaatggtgcgcctgctggaagatggcgattagaagcttatttaaatggctgttttggcggatgagagaagattttcagcctgatacagattaaatcagaacgcagaagcg
gtctgataaaacagaatttgcctggcggcagtagcgcggtggtcccacctgaccccatgccgaactcagaagtgaaacgccgtagcgccgatggtagtgtggggtctccccatgcgagagtagggaactgcc
aggcatcaaataaaacgaaaggctcagtcgaaagactgggcctttcgttttatctgttgtttgtcggtgaacgctctcctgagtaggacaaatccgccgggagcggatttgaacgttgcgaagcaacggcccgga
gggtggcgggcaggacgcccgccataaactgccaggcatcaaattaagcagaaggccatcctgacggatggcctttttgcgtttctacaaactctttttgtttatttttctaaatacattcaaatatgtatccgctcatg
aagcggccgctcatgacgaataaatacctgtgacggaagatcacttcgcagaataaataaatcctggtgtccctgttgataccgggaagccctgggccaacttttggcgaaaatgagacgttgatcggcacgta
agaggttccaactttcaccataatgaaataagatcactaccgggcgtattttttgagttatcgagattttcaggagctaaggaagctaaaatggagaaaaaaatcactggatataccaccgttgatatatcccaatg
gcatcgtaaagaacattttgaggcatttcagtcagttgctcaatgtacctataaccagaccgttcagctggatattacggcctttttaaagaccgtaaagaaaaataagcacaagttttatccggcctttattcacattct
tgcccgcctgatgaatgctcatccggaattccgtatggcaatgaaagacggtgagctggtgatatgggatagtgttcacccttgttacaccgttttccatgagcaaactgaaacgttttcatcgctctggagtgaatac
cacgacgatttccggcagtttctacacatatattcgcaagatgtggcgtgttacggtgaaaacctggcctatttccctaaagggtttattgagaatatgtttttcgtctcagccaatccctgggtgagtttcaccagttttga
tttaaacgtggccaatatggacaacttcttcgcccccgttttcaccatgggcaaatattatacgcaaggcgacaaggtgctgatgccgctggcgattcaggttcatcatgccgtttgtgatggcttccatgtcggcaga
atgcttaatgaattacaacagtactgcgatgagtggcagggcggggcgtaattttttttaaggcagttattggtgcccttaaacgcctggtgctacgcctgaataagtgataataagcggatgaatggcagaaattcg
aaagcaaattcgacccggtcgtcggttcagggcagggtcgttaaatagccgcttatgtctattgctggtttaccggtttattgactaccggaagcagtgtgaccgtgtgcttctcaaatgcctgaggccagttaa
